# Fenofibrate alleviates the brain edema after traumatic brain injury by enhancing endothelial OXPHOS

**DOI:** 10.1016/j.redox.2026.104240

**Published:** 2026-05-27

**Authors:** Qiyuan Feng, Yingwei Gao, Xiaokun Gu, Xinxin Zhao, Xinwen Liu, Zixuan Ma, Wenlan Qi, Zhenghui He, Yuhan Han, Jian Zhang, Wenye Wang, Zhifan Li, Daiwen Zhang, Jialin Huang, Yong Lin, Jiyuan Hui, Bayasgalan Onondari, Galbadrakh Erdenetsetseg, Qing Mao, Jiyao Jiang, Yan Zhou, Weiji Weng, Yan Zhang, Junfeng Feng

**Affiliations:** a Brain Injury Centre, Department of Neurosurgery, Ren Ji Hospital, Shanghai Jiao Tong University School of Medicine, Shanghai, 200127, China; b Shanghai Institute of Head Trauma, Shanghai, 200127, China; c Department of Radiology, Ren Ji Hospital, Shanghai Jiao Tong University School of Medicine, Shanghai, 200127, China; d Shanghai Jiao Tong University School of Medicine, Shanghai, 200025, China; e Department of Brain and Craniofacial Surgery, National Trauma and Orthopaedic Research Center of Mongolia, Ulaanbaatar, 16092, Mongolia; f Administrative Office, National Trauma and Orthopaedic Research Center of Mongolia, Ulaanbaatar, 16092, Mongolia; g The Second Affiliated Hospital, Jiangxi Medical College, Nanchang University, Nanchang, Jiangxi, 330006, China; h Jiangxi Academy of Medical Sciences, Nanchang, Jiangxi, 330006, China; i Nanchang Cranio-Cerebral Trauma Laboratory, Nanchang, Jiangxi, 330006, China

**Keywords:** Traumatic brain injury (TBI), Fenofibrate (FFB), Brain edema, Oxidative phosphorylation (OXPHOS)

## Abstract

Traumatic brain injury (TBI) frequently leads to brain edema, a life-threatening complication and a major cause of mortality and disability. Here, we report that fenofibrate (FFB), an agonist of the nuclear receptor peroxisome proliferator-activated receptor-α (PPARα), alleviates post-traumatic brain edema by enhancing blood-brain barrier (BBB) integrity in a mouse model of severe TBI. Mechanistic studies in cultured endothelial cells and endothelial-specific *PPARα* conditional knockout mice revealed that FFB activates PPARα, enhances mitochondrial oxidative phosphorylation (OXPHOS), and thereby reduces oxidative stress and endothelial cell apoptosis. These findings identify PPARα-dependent enhancement of energy metabolism in the brain endothelium as a promising therapeutic strategy for TBI-induced edema.

## 1 Introduction

Brain edema and the consequent increase in intracranial pressure (ICP) pose significant clinical challenges in the management of patients with traumatic brain injury (TBI) [[1], [2], [3]]. Post-traumatic brain edema predominantly originates from two sources: vasogenic edema and cytotoxic edema. Vasogenic edema is attributed to the disruption of the blood-brain barrier (BBB), resulting in the accumulation of extracellular fluid within the brain parenchyma [4,5]. Conversely, cytotoxic edema is characterized by the accumulation of intracellular fluid, which arises from impaired energy metabolism, inflammatory responses, ion channel dysfunction, etc [[6], [7], [8], [9], [10], [11]]. In the initial stages following TBI, cytotoxic edema is predominant at the site of injury [12]. However, as the integrity of the BBB progressively declines, vasogenic edema emerges as the primary contributor [12,13]. Despite extensive research efforts, the intricate molecular mechanisms underlying post-traumatic brain edema remain inadequately understood.

The current therapeutic strategies endorsed by the Brain Trauma Foundation's Guidelines (such as hyperosmolar therapy, decompressive craniectomy, sedation, etc.) predominantly focus on reducing ICP rather than addressing the specific pathophysiological mechanisms responsible for brain edema formation [14,15]. Consequently, the advancement of therapeutic agents that directly modulate the fundamental processes of edema formation is of substantial clinical importance and therapeutic promise.

Here, we demonstrated that fenofibrate (FFB) activated the peroxisome proliferator-activated receptor alpha (PPARα) in injured cerebrovascular endothelial cells (ECs), leading to the enhancement of mitochondrial oxidative phosphorylation (OXPHOS). This activation effectively inhibited apoptosis of ECs, thereby enhancing the integrity of the BBB and mitigating the progression of brain edema following TBI.

## 2 Results

### 2.1 FFB attenuates brain edema after TBI

**Fig. 1:**
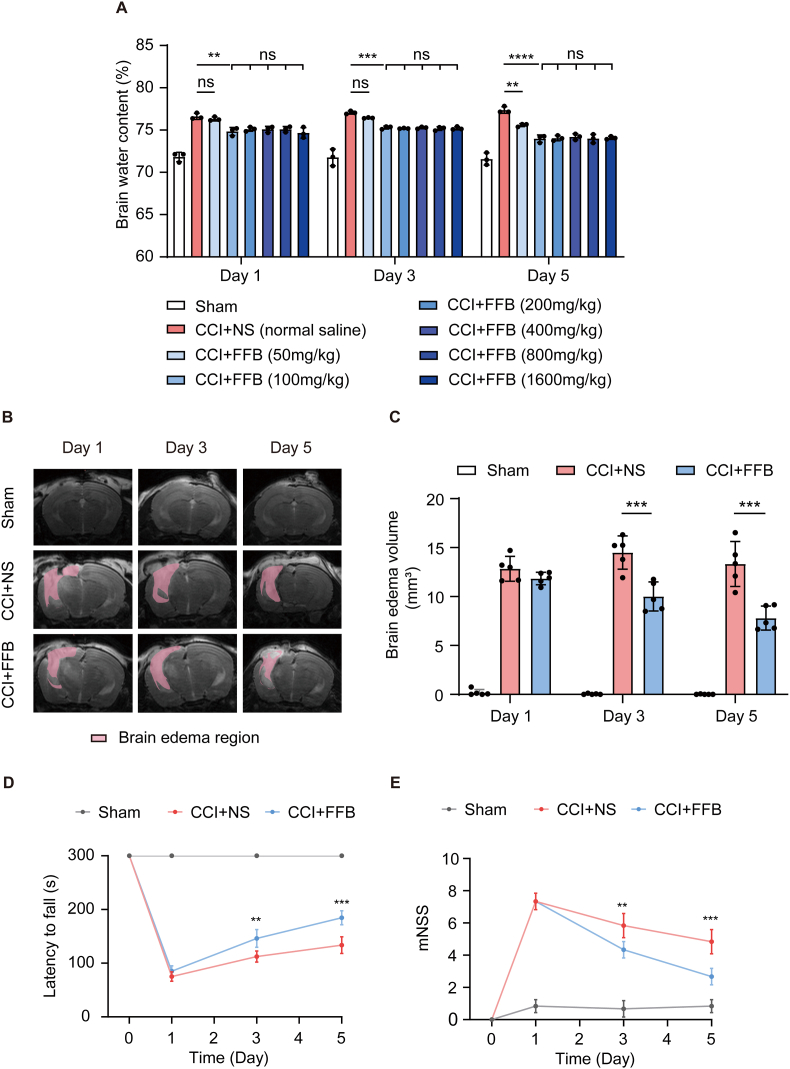
**FFB attenuates brain edema and improves neurological function after CCI.** (**A**) Brain water content measured by the dry/wet weight measurement on Day 1, 3, and 5 after CCI in mice treated with normal saline (vehicle control) or FFB at doses of 50-1600 mg/kg (**B** and **C**) Representative brain edema regions (pink) on MRI (B) and corresponding brain edema volumes from MRI (C) in the sham, CCI with normal saline, and CCI with FFB (100 mg/kg) groups on Day 1, 3, and 5 after CCI. (**D** and **E**) Rotarod (D) and mNSS (E) tests in the sham, CCI with normal saline, and CCI with FFB groups on Day 1, 3, and 5 after CCI. Error bars show mean ± SD. Statistical analysis was performed using one-way ANOVA (A, C) or two-way ANOVA with Tukey's multiple comparison test in (D, E). ns, not significant; **, *p* < 0.01; ***, *p* < 0.001; ****, *p* < 0.0001.

Brain edema is a major contributor to morbidity after traumatic brain injury (TBI), and effective pharmacological options remain limited. Given the roles of PPARα signaling in regulating endothelial metabolism and oxidative stress, we hypothesized that fenofibrate (FFB) might mitigate post-traumatic brain edema. The literature on FFB in experimental TBI is limited, with only a small number of studies published between 2005 and 2008 reporting neuroprotective effects (e.g., anti-inflammatory and anti-oxidative actions) in rodent models [[16], [17], [18]]. To date, no clinical studies have evaluated FFB for mitigating brain edema in TBI patients. Therefore, to test the hypothesis that PPARα activation by FFB can attenuate edema post-TBI, we employed a severe controlled cortical impact (CCI) model in mice. As expected, CCI mice exhibited significantly reduced latency to fall and markedly increased modified Neurological Severity Score (mNSS) scores, validating successful model establishment. (Fig. S1A and B). Given the potential for pharmacokinetic differences and toxicity at high doses, a range of FFB concentrations (50-1600 mg/kg) was evaluated to identify the minimal effective dose in vivo. Oral administration commenced on the day of injury and continued once daily via gavage. Brain water content was assessed using the dry/wet weight measurement on Day 1, 3, and 5 after CCI [19,20]. The results showed that FFB treatment could significantly reduce brain water content after CCI compared to the vehicle control (normal saline), with maximal efficacy observed at a dose of 100 mg/kg (Fig. 1A). In subsequent experiments, CCI mice were treated with FFB at a dose of 100 mg/kg based on the above findings.

To obtain a more dynamic and precise evaluation of FFB's effect on brain edema, cranial magnetic resonance imaging (MRI) was performed on mice on Day 1, 3, and 5 after CCI. Brain edema regions was defined as high signal regions on T2-weighted imaging (T2WI) without corresponding no signal regions on diffusion-weighted imaging (DWI), based on a validated approach for delineating edema boundaries [[21], [22], [23]] (Fig. S2A). Consistent with the results of dry/wet weight measurements, MRI analysis showed that FFB decreased brain edema volumes on Day 3 and 5 after CCI (Fig. 1B and C). Specifically, FFB reduced brain edema volumes to 71% of the vehicle control level on Day 3 and to 61% on Day 5, suggesting that FFB could alleviate brain edema following CCI (Fig. 1C). The apparent diffusion coefficient (ADC) value is a valuable tool for characterizing brain edema on MRI. Vasogenic edema, with its increased extracellular fluid, is typically associated with increased ADC value, while cytotoxic edema, with its cellular swelling, is associated with decreased ADC values [[24], [25], [26]]. We measured the ADC values in the brain edema regions of CCI mice and found that on Day 1 after CCI, the ADC values in the edema regions were comparable to those in the contralateral uninjured regions (Fig. S2B). However, on Day 3 and 5 after CCI, the ADC values in the edema regions were significantly higher than those in the contralateral side (Fig. S2B). These results suggested that vasogenic edema was predominant on Day 3 and 5 after CCI, indicating that FFB could alleviate vasogenic edema. Furthermore, on Day 3 and 5 after CCI, the prolonged latency to fall in the rotarod test and the reduced mNSS scores indicated that FFB improved neurological function in CCI mice, further supporting its role in alleviating brain edema (Fig. 1D and E).

Taken together, these findings demonstrate that FFB treatment can attenuate brain edema after TBI.

### 2.2 FFB enhances blood-brain barrier integrity after TBI

**Fig. 2:**
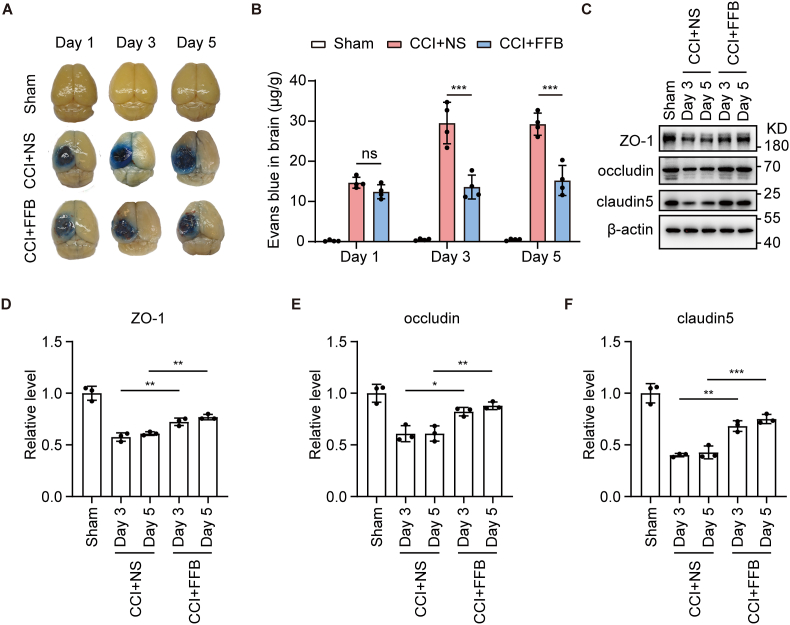
**FFB enhances BBB integrity after CCI.** (**A** and **B**) Representative whole-brains images showing EB extravasation (A) and EB content (B) in sham, CCI with normal saline, and CCI with FFB groups on Day 1, 3, and 5 after CCI. (**C–F**) Representative western blotting showing ZO-1, occludin and claudin-5 expression in perilesional tissue in sham, CCI with normal saline, and CCI with FFB groups on Day 3 and 5 after CCI (C). Quantification of ZO-1 (D), occludin (E) and claudin-5 (F) in western blotting. Error bars show mean ± SD. Statistical analysis was performed using one-way ANOVA with Tukey's multiple comparison test in (B, D-F). ns, not significant; *, *p* < 0.05; **, *p* < 0.01; ***, *p* < 0.001.

As mentioned above, vasogenic edema primarily results from disruption of the BBB. Therefore, we next investigated whether FFB alleviated brain edema by improving BBB integrity. Evans blue (EB) extravasation was used to evaluate BBB permeability [11]. We observed that on Day 1, 3, and 5 after CCI, FFB significantly decreased EB extravasation compared to the vehicle control, indicating that FFB could improve BBB permeability (Fig. 2A and B). We further examined the expression of tight junction proteins, including ZO-1, occludin, and claudin-5, which serve as biomarkers reflecting junction integrity of the endothelial cells (ECs) and, to some extent, the structural integrity of the BBB. Western blotting showed that CCI significantly reduced the expression levels of ZO-1, occludin, and claudin-5 in the perilesional tissue on Day 3 and 5 after injury (Fig. 2C–F). However, compared to the vehicle control, FFB treatment markedly upregulated the expression of these proteins, suggesting that FFB improved the structural integrity of the BBB (Fig. 2C–F). Therefore, these results altogether indicate that FFB treatment enhances the BBB integrity after TBI.

### 2.3 Endothelial PPARα is essential for the brain edema-relieving effect of FFB

**Fig. 3:**
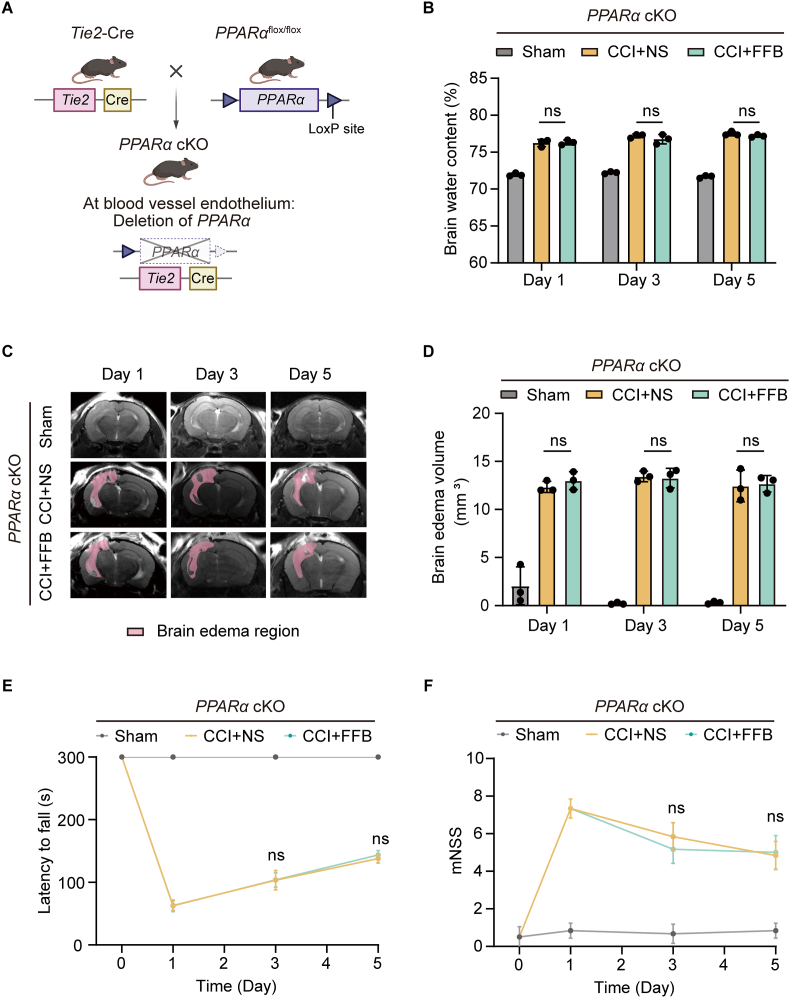
**FFB fails to attenuate brain edema in *PPARα* cKO mice.** (**A**) Schematic illustration of endothelial-specific *PPARα* conditional knockout (*PPARα* cKO) mice generated by crossing *Tie2*-Cre mice with *PPARα*^flox/flox^ mice. (**B**) Brain water content in the sham, CCI with normal saline, and CCI with FFB groups on Day 1, 3, and 5 after CCI in *PPARα* cKO mice. (**C** and **D**) Representative brain edema regions (pink) on MRI (C) and corresponding brain edema volumes (D) in the sham, CCI with normal saline, and CCI with FFB groups on Day 1, 3, and 5 after CCI in *PPARα* cKO mice. (**E** and **F**) Rotarod (E) and mNSS (F) tests in the sham, CCI with normal saline, and CCI with FFB groups on Day 1, 3, and 5 after CCI in *PPARα* cKO mice. Error bars show mean ± SD. Statistical analysis was performed using one-way ANOVA (B, D) or two-way ANOVA with Tukey's multiple comparison test in (E, F). ns, not significant.

Given that FFB enhanced tight junction between ECs, we further explored the molecular mechanisms underlying its effects in ECs. Since FFB is a known PPARα agonist, we first investigated whether it alleviated brain edema through activation of PPARα in ECs. We generated a conditional PPARα knockout line (*PPARα*^flox/flox^; *Tie2*-Cre, *PPARα* cKO) by crossing a conditional *PPARα*^flox/flox^ strain with a *Tie2*-Cre line (Fig. 3A). The protein and mRNA levels of PPARα were markedly reduced in cerebrovascular ECs of the *PPARα* cKO mice, as shown by immunofluorescence staining and qPCR analysis of CD31^+^ ECs isolated from brain tissue via fluorescence-activated cell sorting (FACS) (Fig. S3A–D). The *PPARα* cKO mice and their littermate controls were subjected to dry/wet weight measurements and cranial MRI. No significant differences were observed between genotypes in either assay, indicating that PPARα deletion in ECs did not lead to brain edema (Fig. S4A and B).

Similarly, we assessed brain edema in *PPARα* cKO mice after injury using the dry/wet weight measurements and cranial MRI. Compared to the vehicle control, FFB did not significantly reduce brain water content in *PPARα* cKO mice on Day 1, 3, and 5 after CCI (Fig. 3B). Consistently, FFB also failed to significantly reduce brain edema volume on Day 1, 3, and 5 after CCI in these mice (Fig. 3C and D). In both the rotarod and mNSS tests, no significant differences were observed between the FFB-treated and vehicle-treated groups, further supporting the lack of an effect of FFB on brain edema in *PPARα* cKO mice after CCI (Fig. 3E and F). These results suggest that the brain edema-relieving effect of FFB is dependent on PPARα in ECs.

**Fig. 4:**
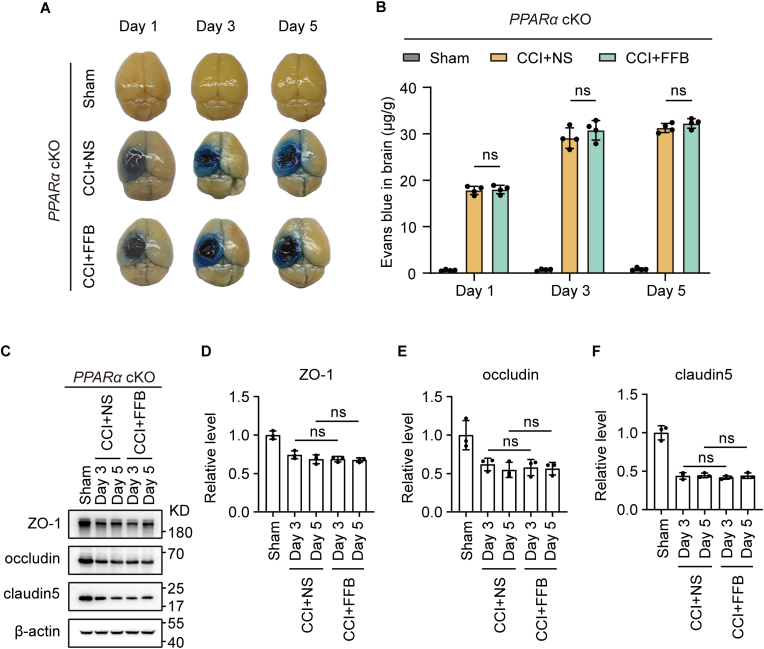
**FFB fails to improve BBB integrity in *PPARα* cKO mice.** (**A** and **B**) Representative whole-brains images showing EB extravasation (A) and EB content (B) in sham, CCI with normal saline, and CCI with FFB groups on Day 1, 3, and 5 after CCI. (**C–F**) Representative western blotting showing ZO-1, occludin and claudin-5 expression in perilesional tissue in sham, CCI with normal saline, and CCI with FFB groups on Day 3 and 5 after CCI (C). Quantification of ZO-1 (D), occludin (E) and claudin-5 (F) in western blotting. Error bars show mean ± SD. Statistical analysis was performed using one-way ANOVA with Tukey's multiple comparison test in (B, D-F). ns, not significant.

Furthermore, we examined whether FFB could still enhance BBB integrity in *PPARα* cKO mice after injury. As expected, no significant differences in EB extravasation were observed between the FFB-treated group and the vehicle control group on Day 1, 3, and 5 after CCI (Fig. 4A and B). Similarly, there were no detectable differences in the protein expression levels of ZO-1, occludin, and claudin-5 between the two groups at these time points (Fig. 4C–F). These results further indicate that the protective effect of FFB on BBB integrity is dependent on endothelial PPARα. Consistent results were obtained using an independent AAV-mediated endothelial-specific PPARα knockout model (AAV-BI30-miniBEND-Cre; Figs. S5–6).

Collectively, the above data suggest that the therapeutic effect of FFB is mediated through the activation of PPARα in ECs.

### 2.4 FFB enhances mitochondrial OXPHOS in ECs

**Fig. 5:**
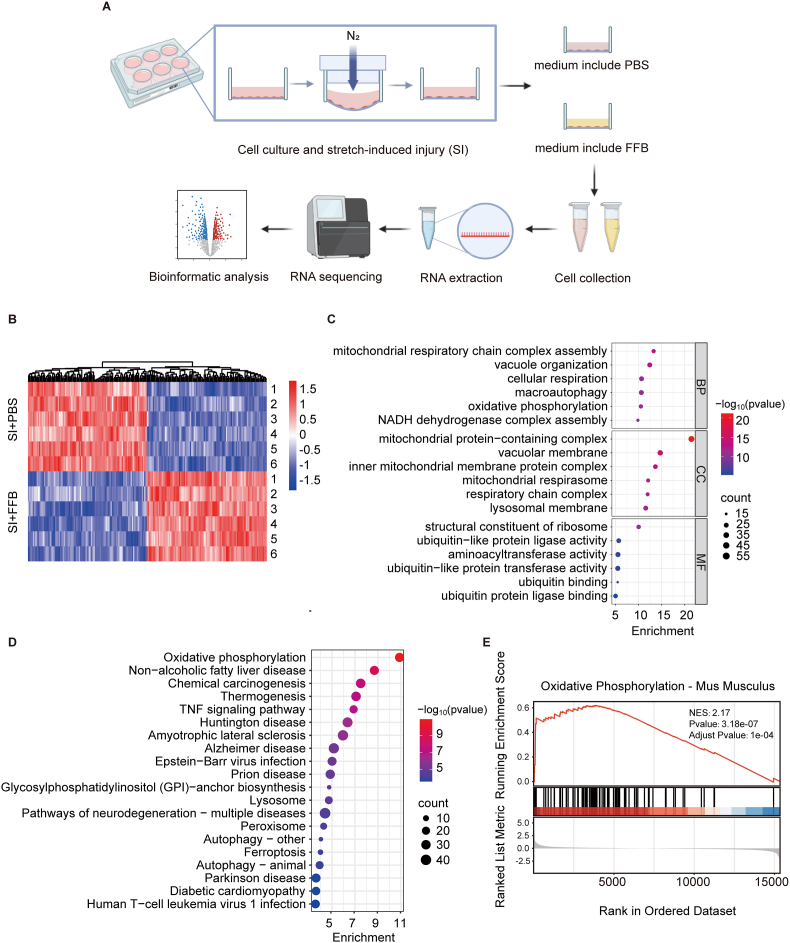
**Transcriptomic profiling reveals enhanced OXPHOS in FFB-treated bEnd.3 cells following injury.** (**A**) Schematic workflow of the RNA sequencing. bEnd.3 cells were subjected to stretch-induced injury (SI), followed by treatment with FFB (100 μM) or vehicle (PBS) for 24 h. Cells were then collected for transcriptomic profiling and bioinformatic analysis. (**B**) Heatmap showing the top 100 upregulated and top 100 downregulated genes between FFB-treated cells and PBS-treated controls. (**C**) Gene ontology (GO) enrichment analysis of the upregulated genes in FFB-treated cells. (**D**) KEGG pathway analysis of the upregulated genes in FFB-treated cells. (**E**) Gene set enrichment analysis (GSEA) of OXPHOS in FFB-treated cells.

To further investigate the molecular mechanisms downstream of PPARα activation by FFB, we established a severe stretch-induced injury (SI) model in bEnd.3 cells, a murine brain vascular endothelial model widely used for BBB studies, to mimic the damage to cerebrovascular ECs following TBI. The shattered cell morphology and the raised lactate dehydrogenase (LDH) levels in the culture medium served as clear indicators of the severe mechanical damage inflicted upon the bEnd.3 cells (Fig. S7A and B). Immediately after injury, cells were treated with FFB (100 μM) or PBS for 24 h. Subsequently, the cells were collected and lysed for transcriptomic profiling by RNA sequencing (Fig. 5A). The heatmap showed top 100 upregulated genes and downregulated genes when comparing FFB-treated cells with PBS-treated cells (Fig. 5B). Gene ontology (GO) analysis of the upregulated genes indicated enrichment in mitochondrial metabolism, including mitochondrial respiratory chain complex assembly and OXPHOS (Fig. 5C). Kyoto encyclopedia of genes and genomes (KEGG) analysis identified OXPHOS as the most significantly enriched pathway following FFB treatment (Fig. 5D). Further examination by gene set enrichment analysis (GSEA) using the OXPHOS activity signature revealed a robust upregulation of OXPHOS by FFB (Fig. 5E). The results from the SI model in bEnd.3 cells indicated that FFB treatment significantly upregulated mitochondrial OXPHOS.

**Fig. 6:**
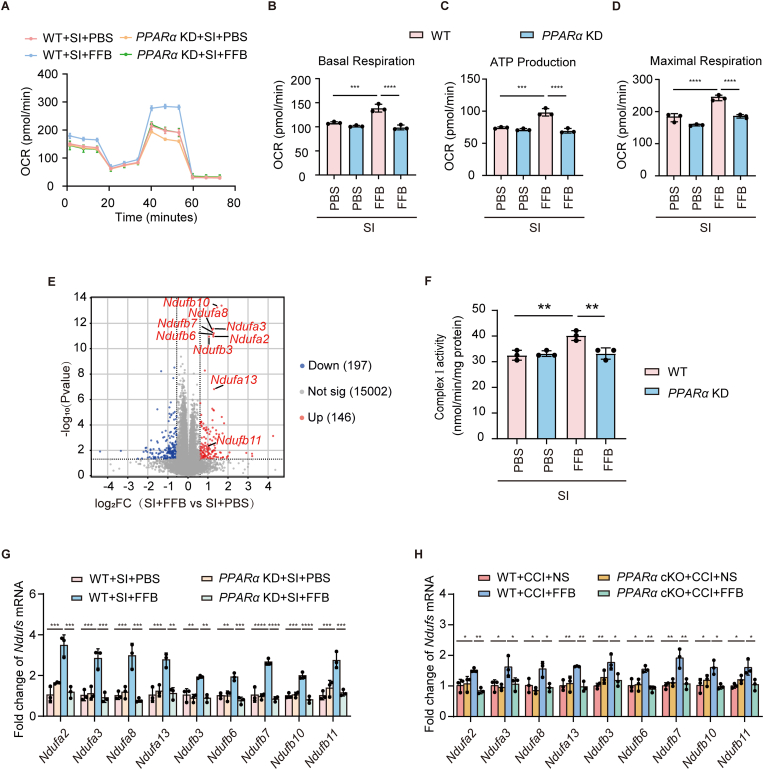
**FFB enhances OXPHOS in ECs.** (**A-D**) OCR analysis in WT and *PPARα* KD bEnd.3 cells following SI with or without FFB treatment. Representative OCR trace (A); Quantification of basal respiration (B), ATP production (C), and maximal respiratory capacity (D). (**E**) Volcano plot showing differentially expressed genes between FFB-treated cells and PBS-treated controls following injury. *Nduf* genes were highlighted. (**F**) Mitochondrail complex I activity in WT and *PPARα* KD bEnd.3 cells following SI with or without FFB treatment. (**G**) Gene expression of *Ndufa* and *Ndufb* measured by qPCR in WT and *PPARα* KD bEnd.3 cells following SI with or without FFB treatment. (**H**) Gene expression of *Ndufa* and *Ndufb* in endothelial cells isolated from the ipsilateral hemispheres of WT and *PPARα* cKO mice, treated with or without FFB, on Day 5 after CCI. Error bars show mean ± SD. Statistical analysis was performed using one-way ANOVA with Tukey's multiple comparison test in (B-D, F–H). *, *p* < 0.05; **, *p* < 0.01; ***, *p* < 0.001; ****, *p* < 0.0001.

To confirm the effect of FFB on OXPHOS, stable *PPARα* knockdown (*PPARα* KD) bEnd.3 cell lines were established and validated by western blotting and qPCR (Fig. S8A–C). Oxygen consumption rates (OCR) were measured to assess OXPHOS activity. Prior to injury, *PPARα* KD cells and control cells exhibited comparable basal respiration, ATP production, and maximal respiratory capacity, indicating that PPARα knockdown did not impair basal mitochondrial function (Fig. S8D–G). Following injury, FFB significantly increased basal respiration, ATP production, and maximal respiratory capacity, whereas this enhancement was absent in *PPARα* KD cells (Fig. 6A–D). These results confirmed that FFB enhanced mitochondrial OXPHOS following endothelial injury in a PPARα-dependent manner.

We next investigated differentially expressed genes related to mitochondrial OXPHOS and found that FFB promoted the upregulation of the *Ndufa* and *Ndufb* gene families including *Ndufa2*, *Ndufa3*, *Ndufa8*, *Ndufa13*, *Ndufb3*, *Ndufb6*, *Ndufb7*, *Ndufb10*, and *Ndufb11*, which encoded accessory subunits of mitochondrial respiratory chain complex I (Fig. 6E). Based on this, we examined complex I activity in bEnd.3 cells. Prior to injury, complex I activity was comparable between *PPARα* KD cells and control cells (Fig. S8H). Following injury, FFB significantly increased complex I activity in WT cells compared to PBS-treated WT cells (Fig. 6F). In contrast, in *PPARα* KD cells, complex I activity remained unchanged after treatment with either FFB or PBS (Fig. 6F). We then validated the expression of *Nduf* genes. *PPARα* knockdown did not affect the expression of *Ndufa* and *Ndufb* in unjuried bEnd.3 cells (Fig. S8I). qPCR verified that FFB increased the expression of *Ndufa* and *Ndufb* in injured WT cells, whereas this effect was abolished when *PPARα* was knocked down (Fig. 6G). Furthermore, we validated the FFB-induced upregulation of *Nduf* genes in vivo. Injured ipsilateral hemispheres were harvested and enzymatically digested, and CD31^+^ ECs were isolated by FACS. Consistent with the findings in bEnd.3 cells, there was no difference in *Ndufa* and *Ndufb* expressions between WT and *PPARα* cKO sham mice (Fig. S8J). However, on Day 5 after CCI, *Ndufa* and *Ndufb* expressions were significantly upregulated in FFB-treated WT mice compared to vehicle-treated controls (Fig. 6H). This upregulation was not observed in *PPARα* cKO mice (Fig. 6H). Therefore, these results above supported that FFB enhanced mitochondrial OXPHOS by increasing the expression of *Ndufa* and *Ndufb* genes in ECs.

### 2.5 Complex I is required for FFB-induced enhancement of mitochondrial OXPHOS in ECs

**Fig. 7:**
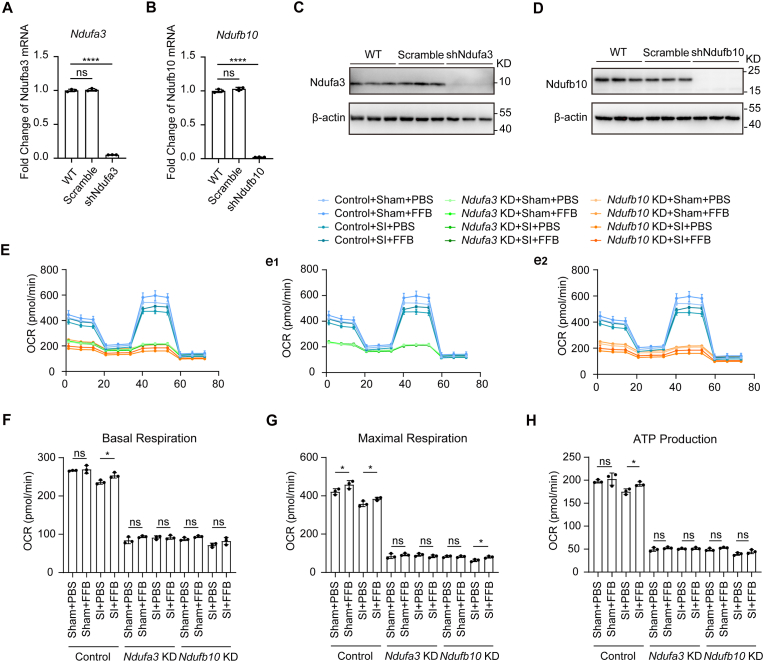
**Knockdown of *Ndufa3* and *Ndufb10* impairs mitochondrial respiration and attenuates the effect of FFB on OXPHOS in bEnd.3 cells.** (**A** and **B**) qPCR analysis showing knockdown efficiency of *Ndufa3* (A) and *Ndufb10* (B) in bEnd.3 cells. (**C** and **D**) Representative western blotting showing Ndufa3 (C) and Ndufb10 (D) expression and corresponding β-actin controls. (**E**, **e1, e2**) Oxygen consumption rate (OCR) measured by Seahorse analysis in control (Scramble), *Ndufa3* KD (sh*Ndufa3*), and *Ndufb10* KD (sh*Ndufb10*) bEnd.3 cells under sham and SI conditions with or without FFB treatment. (**F**–**H**) Quantification of basal respiration (F), maximal respiration (G), and ATP production (H) in bEnd.3 cells under the indicated conditions. Error bars show mean ± SD. Statistical analysis was performed using one-way ANOVA with Tukey's multiple comparison test. ns, not significant; *, *p* < 0.05; ****, *p* < 0.0001.

To determine whether mitochondrial OXPHOS is required for the metabolic effects of FFB, we next disrupted complex I-associated respiration by targeting representative Nduf subunits. Based on our transcriptomic analysis, *Ndufa3* and *Ndufb10* were selected as representative subunits of mitochondrial complex I. Efficient knockdown of *Ndufa3* and *Ndufb10* was confirmed by qPCR and western blotting (Fig. 7A–D).

OCR were then measured to evaluate mitochondrial respiration. In control cells, FFB treatment significantly increased basal respiration, maximal respiratory capacity, and ATP production following SI injury. In contrast, knockdown of either *Ndufa3* or *Ndufb10* markedly reduced overall mitochondrial respiration and attenuated the enhancing effect of FFB on OXPHOS parameters (Fig. 7E–H). Notably, the effect of FFB was almost completely abolished in *Ndufa3*-deficient cells, whereas a partial response to FFB was still observed following *Ndufb10* knockdown.

These results indicate that intact complex I function is required for FFB-mediated enhancement of mitochondrial OXPHOS, with *Ndufa3* potentially playing a more prominent role among the tested subunits.

### 2.6 FFB reduces oxidative stress in ECs in a PPARα-dependent manner

**Fig. 8:**
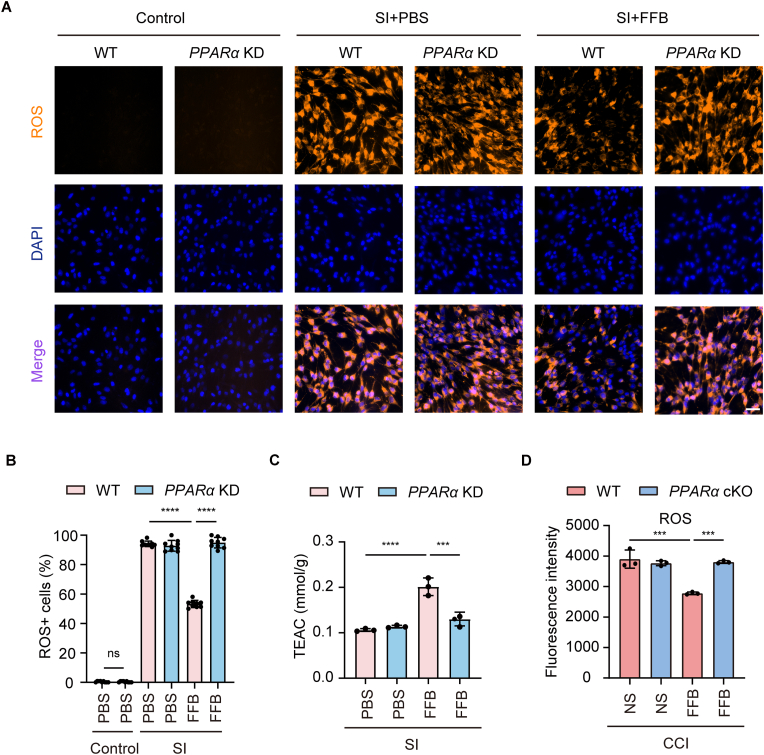
**FFB reduces ROS production in endothelial cells.** (**A**) Representative fluorescence showing ROS (orange) and DAPI (blue) in WT and *PPARα* KD bEnd.3 cells under control conditions, SI with PBS, or SI with FFB, and merged images. Scale bar = 50 μm. (**B**) Quantification of ROS-positive cells in WT and *PPARα* KD bEnd.3 cells under the indicated conditions. (**C**) Trolox equivalent antioxidant capacity (TEAC) of WT and *PPARα* KD bEnd.3 cells following SI treated with or without FFB. (**D**) ROS level in endothelial cells isolated from the ipsilateral hemispheres of WT and *PPARα* cKO mice, treated with or without FFB, on Day 5 after CCI. Error bars show mean ± SD. Statistical analysis was performed using one-way ANOVA with Tukey's multiple comparison test in (B-D). ns, not significant; ***, *p* < 0.001; ****, *p* < 0.0001.

In the mitochondrial respiratory chain, complex I is the first major protein complex involved in electron transfer and one of the primary sites of reactive oxygen species (ROS) generation [27]. Following injury, the structural integrity and function of complex I are impaired, leading to electron accumulation and transfer to oxygen molecules, thereby increasing ROS production [28]. This, in turn, exacerbates oxidative stress and promotes apoptosis. Enhancing mitochondrial OXPHOS may help reduce ROS production at its source. Therefore, we next examined changes in ROS levels following FFB treatment. Intracellular ROS levels were assessed in bEnd.3 cells following SI injury using fluorescence-based detection. ROS accumulation was markedly increased in both WT and PPARα KD cells after injury, indicating injury-induced oxidative stress (Fig. 8A and B). FFB treatment significantly reduced ROS levels in injured WT cells, whereas no such effect was observed in PPARα KD cells (Fig. 8A and B). These findings indicate that FFB attenuates ROS accumulation in endothelial cells following injury in a PPARα-dependent manner. DHE staining, performed as an independent validation, yielded similar results and is presented in Fig. S9A and B. We next performed the trolox equivalent antioxidant capacity (TEAC) assay to evaluate the antioxidant capacity of bEnd.3 cells. TEAC assay results were consistent with DHE staining, supporting the notion that FFB enhanced the antioxidant capacity of injured bEnd.3 cells (Fig. 8C–S8K).

We then examined ROS levels by isolating CD31^+^ ECs from injured ipsilateral hemispheres. Under sham conditions, both *PPARα* cKO mice and their littermate controls displayed similarly low levels of ROS in ECs (Fig. S4C). Following CCI injury, a substantial increase in ROS accumulation was observed in both genotypes. Importantly, FFB significantly attenuated ROS levels in WT mice, whereas such effect was undetected in *PPARα* cKO mice, indicating that FFB reduced endothelial ROS production after TBI in vivo (Fig. 8D).

These results demonstrate that FFB reduces oxidative stress in ECs following injury in a PPARα-dependent manner.

### 2.7 FFB inhibits endothelial apoptosis through mitochondrial OXPHOS

**Fig. 9:**
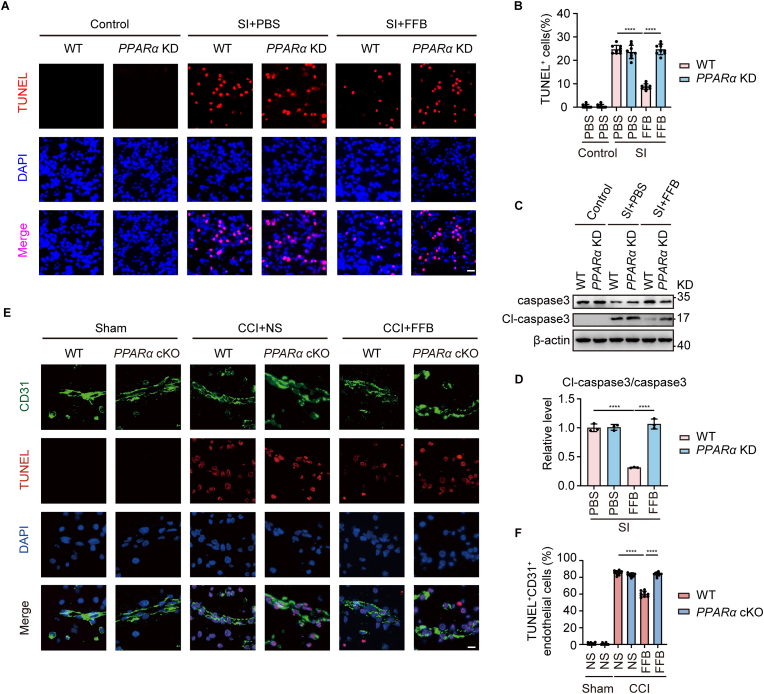
**FFB reduces oxidative stress in ECs in a PPARα-dependent manner** (**A**) Representative TUNEL fluorescence (red) in WT and *PPARα* KD bEnd.3 cells, without SI or following SI, treated with or without FFB as indicated. Scale bar = 50 μm. (**B**) Quantification of TUNEL positive cells in (A). (**C** and **D**) Representative western blotting showing caspase3 and cleaved caspase3 (Cl-caspase3) expression in WT and *PPARα* KD bEnd.3 cells, without SI or following SI, treated with or without FFB as indicated (C). Quantification of Cl-caspase3/caspase3 in western blotting (D). (**E**) Representative immunofluorescence showing co-staining of CD31 (green) and TUNEL (red) in WT and *PPARα* cKO mice, with sham or CCI, treated with or without FFB as indicated. Scale bar = 20 μm. (**F**) Quantification of TUNEL and CD31 positive cells in (E). Error bars show mean ± SD. Statistical analysis was performed using one-way ANOVA with Tukey's multiple comparison test in (B, D, F). ****, *p* < 0.0001.

Finally, we examined the effect of FFB on endothelial apoptosis. In injuried WT bEnd.3 cells, FFB treatment significantly reduced the number of TUNEL-positive cells and the expression of cleaved caspase-3 (Fig. 9A–D). Similarly, in CCI WT mice, the proportion of TUNEL^+^/CD31^+^ cells was markedly decreased following FFB treatment (Fig. 9E and F). As expected, knockdown of PPARα reversed these effects (Fig. 9A–F), indicating that the anti-apoptotic effect of FFB is dependent on PPARα.

**Fig. 10:**
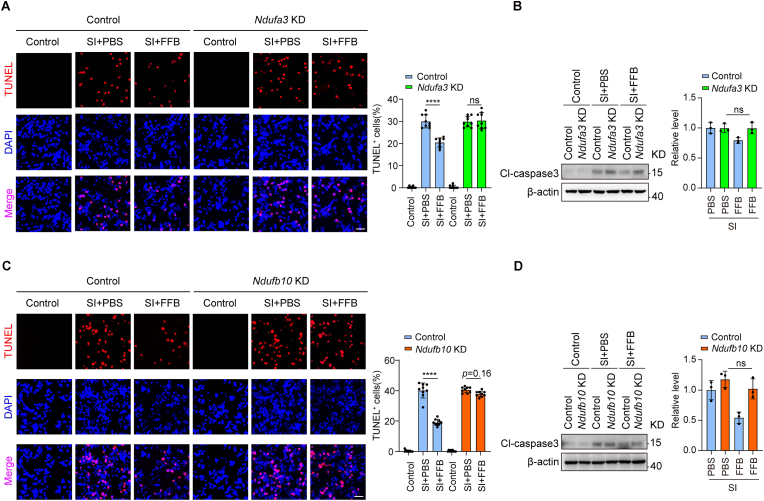
**Knockdown of *Ndufa3* or *Ndufb10* attenuates the anti-apoptotic effect of FFB in bEnd.3 cells under SI conditions.** (**A**) (Left) Representative fluorescence showing TUNEL (red) and DAPI (blue) staining in WT and *Ndufa3* KD bEnd.3 cells under control, SI with PBS, or SI with FFB conditions, scale bar = 50 μm. (Right) Quantification of TUNEL-positive cells. (**B**) (Left) Representative western blotting showing Cl-caspase3 expression in WT and *Ndufa3* KD bEnd.3 cells under the indicated conditions. (Right) Quantification of Cl-caspase3 in western blotting. (**C**) (Left) Representative fluorescence showing TUNEL (red) and DAPI (blue) staining in WT and *Ndufb10* KD bEnd.3 cells under control, SI with PBS, or SI with FFB conditions, scale bar = 50 μm. (Right) Quantification of TUNEL-positive cells. (**D**) (Left) Representative western blotting showing Cl-caspase3 expression in WT and *Ndufb10* KD bEnd.3 cells under the indicated conditions. (Right) Quantification of Cl-caspase3 in western blotting. Error bars show mean ± SD. Statistical analysis was performed using one-way ANOVA with Tukey's multiple comparison test. ns, not significant; ****, *p* < 0.0001.

To further determine whether this anti-apoptotic effect is mediated by mitochondrial OXPHOS, we next examined apoptosis following disruption of complex I. Knockdown of *Ndufa3* almost completely abolished the protective effect of FFB, as evidenced by increased TUNEL-positive cells and cleaved caspase-3 expression. In contrast, a partial anti-apoptotic effect of FFB was still observed in *Ndufb10* KD cells (Fig. 10A–D).

Taken together, these results indicate that mitochondrial OXPHOS, particularly complex I function, is required for the anti-apoptotic effect of FFB in endothelial cells, highlighting PPARα as a key upstream mediator linking metabolic regulation to endothelial protection in the injured brain.

## 3 Discussion

The current study identifies a novel mechanism by which FFB alleviates brain edema after TBI. Specifically, FFB activates PPARα in cerebrovascular ECs following injury, leading to enhanced mitochondrial OXPHOS. This improvement in endothelial mitochondrial function is accompanied by reduced oxidative stress and apoptosis, preservation of BBB integrity, and ultimately attenuation of brain edema.

FFB is a widely used drug for the treatment of hyperlipidemia and has previously been reported to exert neuroprotective effects in rat models of TBI in only three studies [[16], [17], [18]]. These studies demonstrated that FFB could improve neurological function scores [16], suppress certain inflammatory mediators [17], and, in combination with simvastatin, enhance neurological recovery [18]. However, these studies were published nearly two decades ago, employed relatively limited methodologies, and did not investigate the molecular mechanisms underlying FFB's therapeutic effects. Consequently, follow-up work on FFB in the field of TBI has remained limited. Given the central contribution of endothelial dysfunction, oxidative stress, and mitochondrial impairment to post-traumatic brain edema, we investigated whether pharmacological activation of PPARα by FFB could alleviate brain edema after TBI. To evaluate the edema-relieving effect of FFB, we conducted the following experiments. 1) Assessment of brain edema using multiple methods, including the dry/wet weight measurement, MRI, and neurological behavior testing. The dry/wet weight measurement is a traditional approach for evaluating brain edema due to its simplicity. However, it has notable limitations, such as inability to monitor dynamic changes, inability to localize or characterize the edema, and high variability [12,29]. Therefore, we employed complementary methods to provide a more objective and comprehensive evaluation of brain edema in TBI mice. 2) Use of endothelial-specific *PPARα* conditional knockout mice. Since FFB is a PPARα agonist, we tested its effect in mice lacking PPARα in ECs. In these cKO mice, the therapeutic effect of FFB on brain edema was abolished, providing strong evidence that endothelial PPARα is essential for FFB's efficacy. 3) Mechanistic investigation of FFB. Although further mechanistic investigation is warranted, this study provides important initial insights. For example, we primarily focused on the role of ECs in FFB-mediated BBB protection, while other cellular components (such as astrocytes and pericytes) might also contribute significantly. In addition, although we further investigated the involvement of *Nduf* genes in the action of FFB, including knockdown-based functional validation, the detailed regulatory mechanism underlying this process still requires more in-depth investigation. Nonetheless, these mechanistic findings provide additional support for the therapeutic potential of FFB. Together, these three lines of evidence provide strong theoretical support at the animal level for the use of FFB in alleviating brain edema after TBI, laying a foundation for its potential future clinical application in TBI treatment.

Notably, because FFB was administered systemically in vivo, potential peripheral effects should also be considered. Although our genetic and cellular data strongly support an important role of endothelial PPARα in mediating the protective effects of FFB in our model, we cannot fully exclude the possibility that systemic anti-inflammatory actions of FFB may also contribute, at least in part, to the overall neuroprotective phenotype observed in vivo.

Moreover, our study further highlights the critical role of mitochondrial dysfunction and energy metabolism impairment in the pathophysiology of TBI. Mitochondrial dysfunction in TBI is characterized by impaired ATP production, excessive ROS generation, and calcium overload, all of which contribute to cellular injury [30,31]. The burst of ROS leads to oxidative damage of lipids, proteins, and mitochondrial DNA, exacerbating cellular injury [11,30]. Dysfunctional mitochondria also release pro-apoptotic factors such as cytochrome *c*, second mitochondria-derived activator of caspase (Smac), and apoptosis-inducing factor (AIF), which activate the intrinsic apoptotic pathway and promote cell death [32]. In parallel, extrinsic apoptotic signals (such as TNF-α, Fas ligand) further amplify cell death through crosstalk with mitochondrial pathways [32]. Cells attempt to mitigate these insults via autophagy, specifically mitophagy, which removes damaged mitochondria. Mitophagy is generally protective, but if excessively activated under severe stress it can become maladaptive, contributing to cell death [6,33,34]. This complicated interplay of energy failure, oxidative stress, apoptosis, and impaired mitophagy underlies the mitochondrial contribution to acute and chronic neurological abnormality after TBI. In addition, mitochondrial dysfunction has previously been associated with the treatment of brain edema after TBI. Low-dose methylene blue significantly reduced post-traumatic brain edema and lesion volume in preclinical TBI models, while also inhibiting excessive ROS production and preserving mitochondrial function [35,36]. Pharmacological inhibition of mitochondrial ATP synthase with oligomycin B exacerbated post-traumatic cytotoxic edema and elevated intracranial pressure, whereas preservation of mitochondrial function by inhibiting mitochondrial permeability transition pore opening with cyclosporine A significantly attenuated edema and improved mitochondrial integrity [37]. Although these findings have been largely derived from neuronal studies, they primarily reflect general mechanisms of mitochondrial dysfunction after TBI rather than cell-type-specific responses. In contrast, while mitochondrial regulation in brain endothelial cells has been increasingly appreciated, its specific role in TBI-associated BBB dysfunction and edema formation remains insufficiently characterized. Given that endothelial cells form the structural basis of the BBB, their mitochondrial integrity is critical for maintaining barrier stability. In this context, our findings suggest that endothelial mitochondrial dysfunction, particularly impaired OXPHOS, may contribute to BBB disruption and brain edema progression following TBI.

In summary, our study identifies endothelial PPARα-dependent enhancement of mitochondrial OXPHOS as a central mechanism by which FFB preserves BBB integrity and alleviates brain edema following TBI. Building upon previous knowledge of mitochondrial dysfunction in TBI, our findings further extend this concept to the cerebrovascular endothelium, highlighting its critical role in maintaining BBB stability. On one hand, our study proposes FFB as a potential therapeutic strategy for alleviating brain edema after TBI; on the other hand, it identifies endothelial mitochondrial dysfunction as an underappreciated yet promising therapeutic target that may contribute to future TBI treatment approaches.

## 4 Materials and methods

### 4.1 Sex as a biological variable

All in vivo experiments were performed using age-matched male mice (6-8 weeks old). Male mice were chosen to minimize variability introduced by hormonal cycles in females and to ensure experimental consistency. Although only males were used, the biological processes studied are not expected to be sex-specific, and the findings are considered relevant to both sexes.

### 4.2 Experimental animals

C57BL/6 wild-type (WT) mice were purchased from GemPharmatech Co. Ltd. *Tie2*-Cre and *PPARα*^flox/flox^ mice were obtained from Cyagen Biosciences Inc. All procedures involving animal use were approved by the Institutional Animal Care and Use Committee (IACUC) of Ren Ji Hospital, Shanghai Jiao Tong University School of Medicine, and conducted in accordance with relevant institutional and national guidelines. Mice were housed under standard environmental conditions (12-h light/dark cycle, temperature 22 ± 2 °C, humidity 40-60%) with free access to food and water. Prior to experimentation, mice were allowed to acclimate to the housing environment. *In vivo* experiments were performed using age-matched male mice (6-8 weeks old), which were randomly assigned to experimental groups. Investigators were blinded to group allocation during data collection and analysis to ensure experimental rigor.

### 4.3 Postnatal lateral ventricle injection of AAV

To achieve postnatal endothelial-specific deletion of *PPARα*, *PPARα*^flox/flox^ neonatal mice at postnatal day 0-4 (P0–P4) were cryoanesthetized on ice for 2-3 min. AAV-BI30-miniBEND-Cre (AAV-EC-Cre; Brain Case, China) was injected into the lateral ventricles using a 5 μL Hamilton microsyringe. The needle was advanced to a depth of 3 mm, and 2 μL of viral suspension was delivered into each lateral ventricle. After injection, the needle was kept in place for 1 min before withdrawal. The injection site was then wiped with 75% ethanol, and pups were marked and returned to the cage for further rearing. Two months after injection, mice were used for subsequent experiments.

### 4.4 Controlled cortical impact (CCI) model

Adult male mice were subjected to controlled cortical impact (CCI) injury as previously [6,38,39]. Mice were anesthetized with 4% isoflurane in oxygen and fixed in a stereotaxic apparatus. A craniotomy was performed 2.5 mm lateral to the left sagittal suture at the level of the bregma. Cortical injury was induced using a controlled cortical impactor (YHCI99; Wuhan Yihong Technology Co., Ltd.) with a 3 mm diameter impactor tip, at an impact depth of 2.5 mm, velocity of 6.0 m/s, and dwell time of 100 ms. Sham-operated mice underwent the same procedure without cortical impact.

### 4.5 Dry/wet weight measurement

Brain water content was determined by dry/wet weight measurement as previously described [40]. Mice were anesthetized and transcardially perfused with cold phosphate-buffered saline (PBS) to remove intravascular blood. Brains were quickly removed and weighed to obtain the wet weight. Samples were then dried at 100 °C for 72 h and reweighed to determine the dry weight. Brain water content was calculated as follows: Brain water content (%) = (wet weight − dry weight)/wet weight × 100%.

### 4.6 Magnetic resonance imaging (MRI)

Brain edema volume was assessed using T2-weighted imaging (T2WI) and diffusion-weighted imaging (DWI) sequences on a 7.0T small-animal MRI system (BioSpec 70/20, Bruker, Germany). Imaging parameters for T2WI were: repetition time = 2500 ms, echo time = 36 ms, slice thickness = 3 mm, field of view = 20 × 20 mm^2^, and matrix size = 256 × 256. Apparent diffusion coefficient (ADC) maps were generated based on DWI data. Quantification of brain edema volume and ADC values was performed using RadiAnt DICOM Viewer (v2023.1, Medixant, Poland). To quantify brain edema volume, high signal regions on T2WI were manually delineated for each slice, while corresponding areas with no signal on DWI were excluded to minimize artifacts and improve specificity for edema. The edema area per slice was calculated by subtracting non-edematous regions (based on DWI) from the high signal regions on T2WI. Total edema volume was then obtained by summing the edema areas across all slices and multiplying by the slice thickness.

### 4.7 Behavioral test

Modified neurological severity score (mNSS) and rotarod tests were performed as previously described to evaluate neurological deficits following CCI [41,42]. Mice were pre-trained for 3 consecutive days before injury. For the mNSS test, mice were required to complete three tasks: circle exit, plane walk, and beam walk. Scores ranged from 0 (no deficit) to 10 (severe impairment) based on task performance. For the rotarod test, mice were individually placed and crawled as the rod rotated. The test ended when the mouse fell or reached the maximum duration of 300 s. The falling time was recorded as an indicator of motor coordination and balance.

### 4.8 Evans blue extravasation measurement

Evans blue (EB) extravasation was assessed as previously described with slight modifications [43,44]. Mice were intravenously injected with EB (30 mg/ml in saline) at a dose of 45 mg/kg via the tail vein. After 4 h of circulation, mice were transcardially perfused with ice-cold PBS to remove intravascular dye. Brains were collected and weighed, then homogenized in 1 ml of formamide and incubated at 60 °C for 48 h. The homogenates were centrifuged at 10,000 g for 10 min, and the absorbance of the supernatant was measured at 620 nm using a microplate reader. EB content was quantified based on a standard curve and normalized to brain weight.

### 4.9 Cell culture

bEnd.3 cells (TCM40) and HEK-293T cells (GNHu17) were purchased from Cell Bank/Stem Cell Bank, Chinese Academy of Sciences (Shanghai, China). All types of cells were cultured in DMEM supplemented with 10% FBS and 1% penicillin-streptomycin. Cells were maintained at 37 °C in a humidified incubator with 5% CO_2_. All cell lines were verified by STR profiling at the Cell Bank/Stem Cell Bank, Chinese Academy of Sciences and regularly tested negative for mycoplasma.

### 4.10 Stretch-induced injury model in vitro

TBI was performed using a well-established model of mechanical stretch injury [45]. bEnd.3 cells were seeded onto BioFlex culture plates (BF–3001C; Flexcell International Corporation, USA) and cultured to approximately 80% confluence. Cells were then subjected to stretch injury using a nitrogen-driven Cell Injury Controller II (Custom Design & Fabrication, Inc., USA), as previously described [6]. A single pulse was applied with the following parameters: delay, 50 ms; regulator pressure, 44 psi; and peak pressure, 4 psi. Cells were maintained under standard conditions (37 °C, 5% CO_2_) after injury. The concentration of lactate dehydrogenase (LDH) in the culture supernatant was measured 24 h after stretch-induced injury using an LDH assay kit (C0016; Beyotime, China), following the manufacturer's instructions.

### 4.11 Western blotting

Western blotting was performed as described previously with minor modifications [6,46]. Cells and brain tissues were lysed in SDS lysis buffer (50 mM Tris-HCl, 3% SDS, 5% glycerol, pH 7.5). Lysates were boiled at 95 °C for 10 min, then diluted 5-fold with Triton X-100 lysis buffer (20 mM NEM, 20 mM Tris-HCl, 150 mM NaCl, 1% Triton X-100, 2 mM EDTA, 10% glycerol, pH 7.5) containing protease inhibitor cocktail. After centrifugation at 21,130 × g for 20 min at 4 °C, the supernatant was collected. They were boiled in the SDS sample buffer and then subjected to SDS-PAGE and immunoblotting. Gray values of immunoblotting bands were quantified using ImageJ software (National Institutes of Health, USA). The following primary antibodies were used in this study: anti-ZO-1 (1:1000; 21773-1-AP; Proteintech, USA), anti-claudin-5 (1:1000; A10207; Abclonal, China), anti-occludin (1:1000; 27260-1-AP; Proteintech, USA), anti-caspase-3 (1:1000; T40044; Abmart, China), anti-cleaved caspase-3 (1:1000; 9661; Cell Signaling Technology, USA), anti-β-actin (1:5000; AB2001; Abways, China), and anti-PPARα (1:1000; PA1-822A; Invitrogen, USA). The secondary antibody used was HRP-conjugated goat anti-rabbit IgG (H + L) (1:2000; AS014; Abclonal, China). Antibody incubations were performed according to the manufacturers’ instructions.

### 4.12 RNA sequencing

Total RNA was extracted using the RNA-easy Isolation Kit (R701-01; Vazyme, China) following the manufacturer's protocol. Briefly, cells were lysed in the provided RNA lysis buffer, followed by phase separation using chloroform and centrifugation at 12,000 × g for 10 min at 4 °C. The aqueous phase was collected, and RNA was precipitated with isopropanol at −20 °C for 1 h. After washing with 75% ethanol, RNA pellets were air-dried and resuspended in RNase-free water. Genomic DNA contamination was removed using RNase-free DNase I (AM2238; TURBO DNase, Thermo Fisher Scientific, USA). Polyadenylated RNA was isolated using the Poly(A) mRNA magnetic isolation module (E7490; New England Biolabs, USA). RNA-seq libraries were prepared using the Ultra II directional RNA library prep kit for Illumina (E7760; New England Biolabs, USA), and library quality was assessed using the Agilent Bioanalyzer 2100 system. Paired-end sequencing (2 × 150 bp) was performed on an Illumina platform. Raw reads were processed using Cutadapt (v1.18) and Trimmomatic (v0.35) to remove adapter sequences and low-quality bases. Quality control was conducted using FastQC. Clean reads were aligned to the mouse reference genome (GRCm39) using HISAT2 (v2.1.0), and gene expression levels were quantified using StringTie (v1.3.4d). Differential expression analysis was performed using edgeR (v3.24.2) with false discovery rate (FDR) correction for multiple testing. Differentially expressed genes were identified according to the thresholds indicated in the corresponding figure legends. Functional enrichment analyses, including Gene Ontology (GO), Kyoto Encyclopedia of Genes and Genomes (KEGG), and gene set enrichment analysis, were performed using R-based bioinformatics packages, including clusterProfiler. GO enrichment analysis included Biological Process (BP), Cellular Component (CC), and Molecular Function (MF) categories. Significantly enriched GO terms and KEGG pathways were determined using an adjusted P value threshold of <0.05. For visualization, enriched terms or pathways were ranked according to adjusted P value, and the top enriched results were displayed without manual curation. Heatmaps, volcano plots, enrichment plots, and bubble plots were generated using ggplot2 and related R packages.

### 4.13 Generation of stable knockdown cell lines

To generate stable knockdown (KD) bEnd.3 cell lines, shRNA sequences targeting mouse *PPARα*, *Ndufa3*, or *Ndufb10* were individually cloned into the PLKO.1-3×FLAG-CopGFP-Puro vector (Bioscience, China) using T4 DNA ligase (M0202; New England Biolabs, USA). Lentiviral particles were produced by co-transfecting HEK-293T cells with the shRNA plasmid, psPAX2, and pMD2.G packaging plasmids (5:2.5:1) using NB transfection reagent (Bioscience, China) in Opti-MEM medium. Viral supernatants were collected at 24 and 48 h post-transfection, filtered through a 0.45 μm membrane, and concentrated by ultracentrifugation. bEnd.3 cells at 70% confluence were infected with the viral supernatants in the presence of 8 μg/ml polybrene (TR-1003-G; Sigma-Aldrich, USA) for 12-16 h. After infection, cells were cultured in fresh medium and selected with 15 μg/ml puromycin (ant-pr-1; InvivoGen, USA) to establish stable KD cell lines. Puromycin-resistant colonies were expanded for subsequent experiments.

### 4.14 Respiratory chain complex I activity assay

Mitochondrial complex I activity was assessed using Mitochondrial complex I Activity Assay Kit (BC0515; Solarbio, China) according to the manufacturer's instructions with minor modifications. bEnd.3 cells cultured in 6-well plates were collected and homogenized in ice-cold extraction buffer I. The homogenates were centrifuged at 600 × g for 10 min at 4 °C to remove debris, followed by 11,000 × g for 15 min at 4 °C to isolate mitochondria. The resulting pellet was resuspended in extraction buffers I and II and subjected to ultrasonic disruption on ice. Complex I activity The activity was expressed as ΔA/min, calculated as the difference between the initial (A1) and final (A2) absorbance values at 340 nm during the 1-min measurement period (ΔA = A1 − A2).

### 4.15 Mitochondrial stress test

Mitochondrial oxygen consumption rate (OCR) was measured using the Seahorse XF Cell Mito Stress Test on the XFe96 Analyzer (Agilent Technologies, USA). bEnd.3 cells were seeded into Seahorse XF96 cell culture microplates at a density of 20,000 cells per well and incubated overnight at 37 °C with 5% CO_2_ to reach 80-90% confluence. Intially, the sensor cartridge was hydrated in XF Calibrant at 37 °C in a non-CO_2_ incubator for at least 1 h. Cells were washed and incubated with 180 μL of pre-warmed assay medium (XF Base Medium supplemented with 10 mM glucose, 1 mM pyruvate, and 2 mM glutamine; pH 7.4) at 37 °C in a non-CO_2_ incubator for 1 h prior to measurement. OCR was recorded in response to sequential injections of oligomycin (1 μM), FCCP (1.5 μM), and rotenone/antimycin A (0.5 μM each). Data acquisition and analysis were performed using Wave software (v2.6.3, Agilent Technologies), and results were normalized to total protein content or cell number as appropriate.

### 4.16 Trolox equivalent antioxidant capacity assay

Trolox equivalent antioxidant capacity (TEAC) was measured using an ABTS-based colorimetric assay kit (S0119; Beyotime, China) following the manufacturer's instructions. bEnd.3 cells were harvested and homogenized in 1 ml of ice-cold extraction buffer, then centrifuged at 600 × g for 10 min at 4 °C. The supernatant was collected and further centrifuged at 11,000 × g for 15 min at 4 °C. The resulting pellet was resuspended in 200 μl of extraction buffer I and 200 μl of extraction buffer II, and disrupted by sonication on ice. For the assay, 20 μl of each sample or Trolox standard was mixed with 170 μl of ABTS reagent in a 96-well plate and incubated at 37 °C for 6 min. Absorbance was measured at 414 nm using a microplate reader. TEAC was calculated from a Trolox standard curve and expressed as μmol Trolox equivalents per 10^6^ cells.

### 4.17 Quantitative real-time PCR

Total RNA was extracted using RNA-easy Isolation Reagent (R701-01; Vazyme, China) or the PicoPure™ RNA Isolation Kit (KIT0204; Thermo Fisher Scientific, USA) for low-input samples such as FACS-sorted endothelial cells. Genomic DNA was removed using gDNA wiper mix (R323; Vazyme, China). First-strand cDNA was synthesized using HiScript III qRT SuperMix (R323; Vazyme, China). Quantitative real-time PCR was performed using SGExcel FastSYBR Mixture (B532955; Sangon Biotech, China) on a LightCycler 480 system (Roche, Germany). Gene expression was normalized to β-actin.

### 4.18 Immunofluorescence staining

Immunofluorescence staining was performed on paraffin-embedded mouse brain sections. Sections were deparaffinized in xylene, rehydrated through graded ethanol, and subjected to heat-induced antigen retrieval in EDTA buffer at 92 °C for 48 min. After PBS washes (3 × 5 min, pH 7.4), sections were encircled with a hydrophobic barrier pen and blocked with 3% BSA for 30 min at room temperature. For goat-derived primary antibodies, 10% donkey serum was used as the blocking agent. Sections were incubated overnight at 4 °C with primary antibodies against PPARα (1:300; 66826-1-Ig; Proteintech, USA) or CD31 (1:300; GB113151; Servicebio, China), or with TUNEL reaction mix (G1502; Servicebio, China) in a humidified chamber. The following day, sections were washed and incubated with fluorescently labeled secondary antibodies for 50 min at room temperature in the dark. Nuclei were counterstained with DAPI for 10 min. Autofluorescence was quenched using antifade reagent for 5 min, and slides were mounted with antifade mounting medium. Images were acquired using a Nikon Eclipse C1 fluorescence microscope (Nikon, Japan) and analyzed using SlideViewer software (v2.8.0.216379, 3DHISTECH, Hungary). For intracellular ROS detection, CellROX Orange staining and dihydroethidium (DHE) staining were performed in bEnd.3 cells according to the manufacturers' instructions. Briefly, cells were cultured to approximately 80% confluence, treated as indicated, and then incubated with CellROX Orange working solution (5 μM; 50103ES50, Yeasen, China) or DHE working solution (1:1000; S0064, Beyotime, China) at 37 °C for 30 min. After washing with PBS, cells were counterstained with DAPI for 10 min. Fluorescence images were captured using a Zeiss fluorescence microscope (Carl Zeiss, Germany) and analyzed with ZEN software (v2012, Carl Zeiss, Germany). TUNEL staining of cultured bEnd.3 cells was conducted using the TUNEL BrightRed Apoptosis Detection Kit (A113-02; Vazyme, China), in accordance with the manufacturer's instructions. After labeling, cells were counterstained with DAPI and imaged as described above.

### 4.19 Flow cytometry analysis

Mouse cortical tissue was collected, finely minced, and digested at 37 °C for 45 min in a solution containing collagenase IV (C5138; Sigma-Aldrich, USA) and DNase I (11284932001; Roche, Switzerland). After mechanical dissociation and filtration through a 100 μm mesh, the cell suspension was resuspended in 40% Percoll (17-0891-01; GE Healthcare, USA) and centrifuged at 400 × g for 25 min to enrich for microvascular cells. The pellet was treated with RBC lysis buffer (420301; BioLegend, USA), washed with PBS, and resuspended in staining buffer for downstream applications. For fluorescence-activated cell sorting (FACS), cells were stained with FITC-conjugated anti-CD31 antibody (1:100; 11-0311-82; eBioscience, USA) and eFluor 780 viability dye (65-0865-14; Thermo Fisher Scientific, USA) for 30 min at 4 °C in the dark. After washing and filtration through a 40 μm strainer, live (eFluor 780^-^) CD31^+^ endothelial cells were sorted on a MoFlo XDP cell sorter (Beckman Coulter, USA) in Purity Mode. Sorting gates were established using fluorescence-minus-one (FMO) controls, and collected cells were transferred into tubes containing RPMI-1640 medium supplemented with 10% FBS. For intracellular ROS detection, a separate cell aliquot was pre-treated with Fc receptor blocking reagent (422301; BioLegend, USA), then stained with PeCy7-conjugated anti-CD31 (102418; BioLegend, USA) and a FITC-labeled ROS detection probe (88-5930-74; Thermo Fisher Scientific, USA). After washing, samples were analyzed on a FACSAria III flow cytometer (BD Biosciences, USA). Data were analyzed using FlowJo software (v10.8.1, BD, USA), and CD31^+^ endothelial cells were gated after exclusion of debris and dead cells.

### 4.20 Statistical analysis

Statistical analyses were performed using GraphPad Prism (v9.0, GraphPad Software, USA). Data are presented as mean ± standard deviation (SD). Comparisons between two groups were analyzed using unpaired two-tailed Student's *t*-tests. One-way analysis of variance (ANOVA) followed by Tukey's or Bonferroni post hoc test was used for comparisons among more than two groups. For experiments involving multiple variables, two-way ANOVA was applied with appropriate post hoc corrections. *P* < 0.05 were considered statistically significant. Significance levels were denoted as follows: *p* < 0.05 (*), *p* < 0.01 (**), *p* < 0.001 (***), and *p* < 0.0001 (****). All experiments were performed with at least three biological replicates unless otherwise specified.

### 4.21 Study approval

All animal procedures were approved by the Institutional Animal Care and Use Committee (IACUC) of Ren Ji Hospital, Shanghai Jiao Tong University School of Medicine, and were conducted in accordance with relevant institutional and national guidelines.

## Funding

This study was supported by grants from the 10.13039/501100001809National Natural Science Foundation of China (82501633 to Y.G., 82302147 to X.Z., 82401610 to W.Weng, 82260378 to Y.Zhang, and 82371379 to J.F.), the Leading Talent Program of Shanghai Municipal Health Commission (2022LJ023 to Y.Zhou), Eastern Talent Plan Leading Project (LJ2023127 to Y.Zhou), High-end Talent Program for Science, Technology and Innovation (G3423 to Y.Zhang), and Shanghai Municipal Education Commission-Gaofeng Clinical Medicine Support (02.101005.001.30.30A to J.F.).

## CRediT authorship contribution statement

**Qiyuan Feng:** Data curation, Formal analysis, Investigation, Methodology, Visualization, Writing – review & editing. **Yingwei Gao:** Data curation, Formal analysis, Funding acquisition, Investigation, Visualization, Writing – review & editing. **Xiaokun Gu:** Data curation, Formal analysis, Investigation, Visualization, Writing – review & editing. **Xinxin Zhao:** Data curation, Methodology, Writing – review & editing. **Xinwen Liu:** Formal analysis, Writing – review & editing. **Zixuan Ma:** Formal analysis, Writing – review & editing. **Wenlan Qi:** Formal analysis, Writing – review & editing. **Zhenghui He:** Formal analysis, Writing – review & editing. **Yuhan Han:** Visualization. **Jian Zhang:** Visualization. **Wenye Wang:** Visualization. **Zhifan Li:** Visualization. **Daiwen Zhang:** Visualization. **Jialin Huang:** Visualization. **Yong Lin:** Visualization. **Jiyuan Hui:** Visualization. **Bayasgalan Onondari:** Visualization. **Galbadrakh Erdenetsetseg:** Visualization. **Qing Mao:** Visualization. **Jiyao Jiang:** Visualization. **Yan Zhou:** Funding acquisition, Methodology, Writing – original draft, Writing – review & editing. **Weiji Weng:** Conceptualization, Data curation, Funding acquisition, Visualization, Writing – original draft, Writing – review & editing. **Yan Zhang:** Conceptualization, Funding acquisition, Writing – original draft, Writing – review & editing. **Junfeng Feng:** Conceptualization, Funding acquisition, Writing – original draft, Writing – review & editing.

## Declaration of competing interest

The authors declare that they have no known competing financial interests or personal relationships that could have appeared to influence the work reported in this paper.

## Data Availability

Data will be made available on request.
