# Supplementary material for: Fenofibrate alleviates the brain edema after traumatic brain injury by enhancing endothelial OXPHOS
Source: Redox Biol. 2026 May 27;94:104240. doi: 10.1016/j.redox.2026.104240 (PMC13241661; doi:10.1016/j.redox.2026.104240)
Supplement: Multimedia component 1 [file mmc1.docx]

**Supplementary Materials**


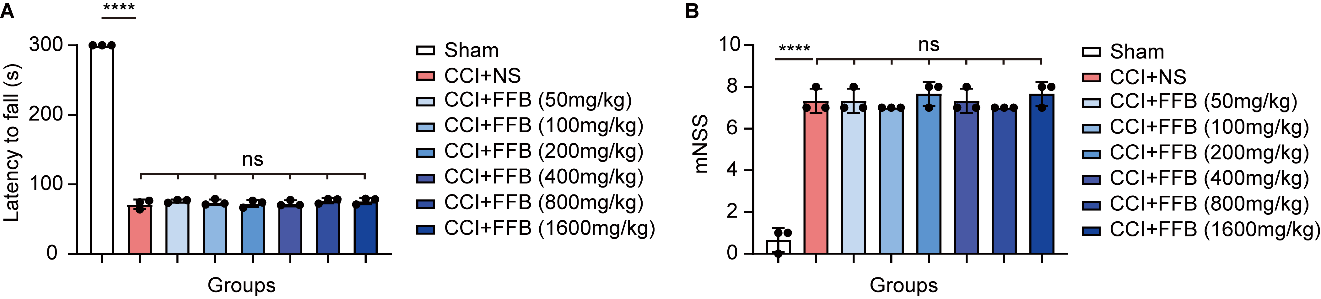


**Figure S1. Establishment of a severe CCI model.** (**A** and **B**) Rotarod (A) and mNSS (B) tests in mice treated with normal saline (vehicle control) or FFB at doses of 50-1600 mg/kg on Day 1 after CCI. Error bars show mean ± SD. Statistical analysis was performed using one-way ANOVA with Tukey’s multiple comparison test (A, B). ns, not significant; ****, *p* < 0.0001.


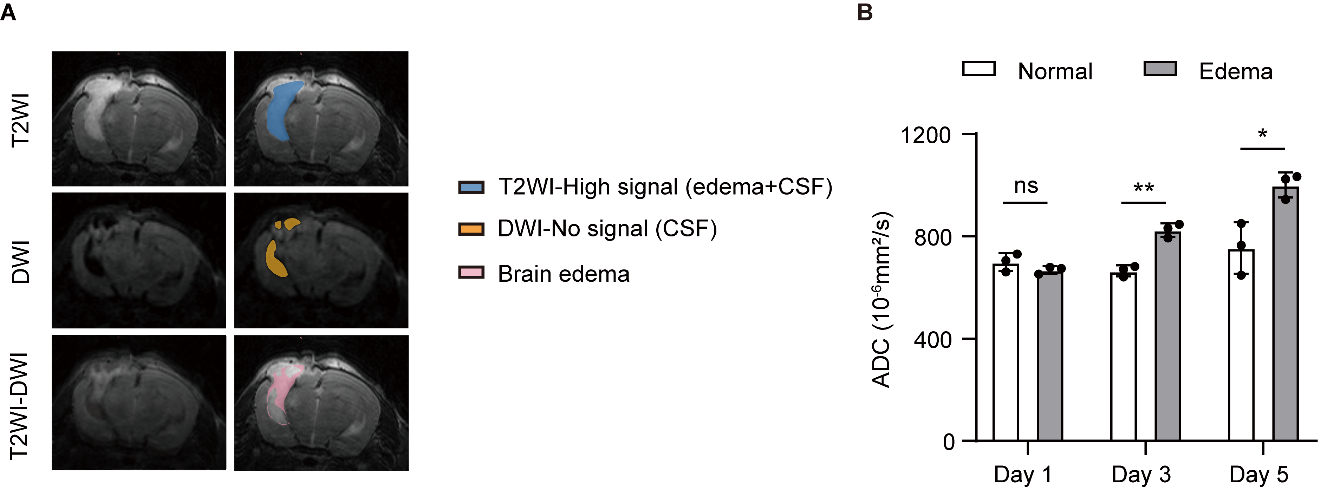


**Figure S2. The brain edema on MRI after CCI.** (**A**) Brain edema regions were defined as areas with high signal on T2-weighted imaging (T2WI, blue) without corresponding no signal regions on diffusion-weighted imaging (DWI, orange). The brain edema areas was identified by pink. (**B**) Apparent diffusion coefficient (ADC) values of the brain edema regions and the contralateral normal regions on Day 1, 3, and 5 after CCI. Error bars show mean ± SD. Statistical analysis was performed using unpaired two-tailed *t*-test in (B). ns, not significant; *, *p* < 0.05; **, *p* < 0.01.


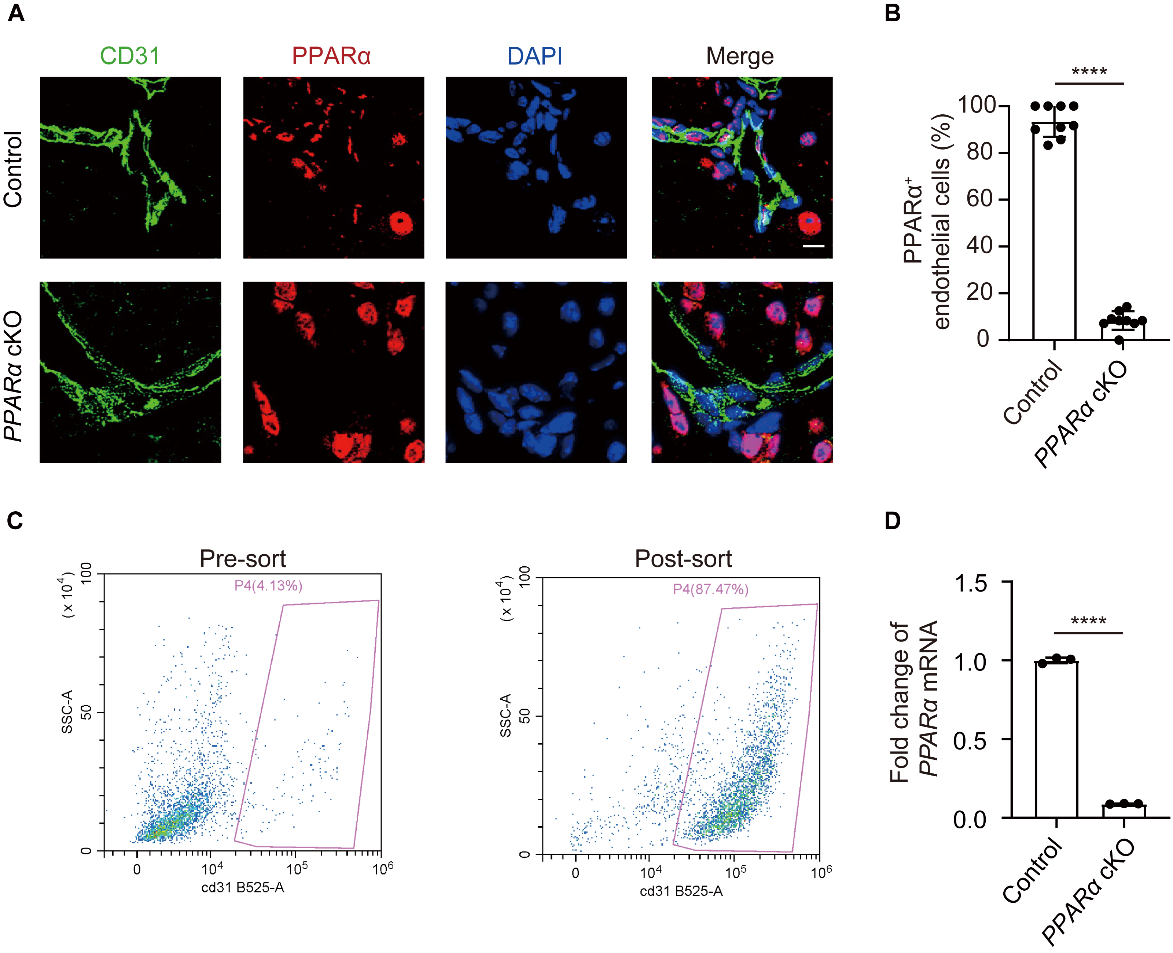


**Figure S3. Validation of endothelial-specific PPARα conditional knockout (*PPARα* cKO) mice.** (**A**) Representative immunofluorescence showing co-staining of CD31 (green) and PPARα (red) in cortical sections from *PPARα* cKO mice and their littermate control. Scale bar = 20 μm. (**B**) Quantification of PPARα and CD31 positive cells in (A). (**C**) Representative flow cytometry plots showing the percentage of CD31 positive cells before and after sorting from brain tissue. (**D**) Gene expression of *PPARα* in endothelial cells isolated from brain tissue of *PPARα* cKO mice and their littermate control. Error bars show mean ± SD. Statistical analysis was performed using unpaired two-tailed *t*-test in (B, D); ****, *p* < 0.0001.


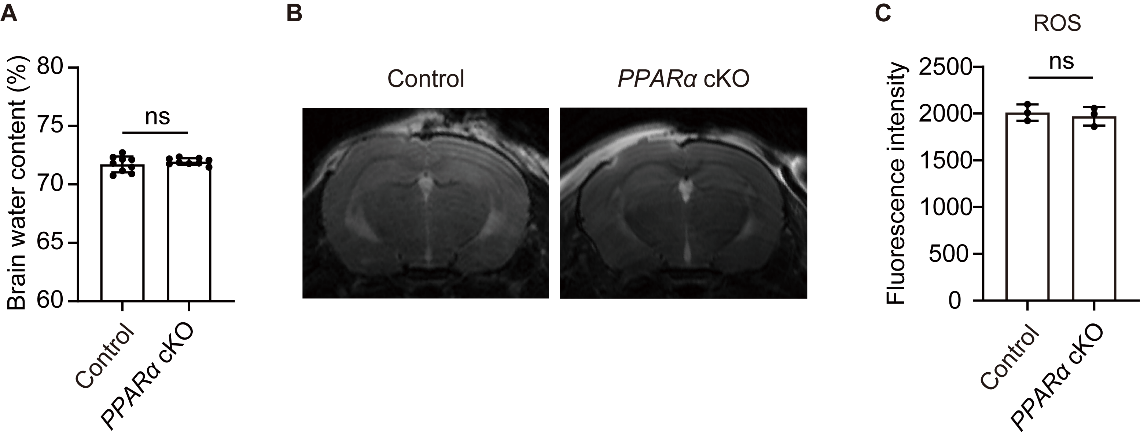


**Figure S4. Endothelial-specific PPARα conditional knockout does not affect brain water content, brain edema, or ROS levels without CCI.** (**A**) Brain water content in *PPARα* cKO mice and their littermate control without CCI. (**B**) Representative crainial MRI in *PPARα* cKO mice and their littermate control without CCI. (**C**) ROS level in endothelial cells isolated from the brain of *PPARα* cKO mice and their littermate control without CCI. Error bars show mean ± SD. Statistical analysis was performed using unpaired two-tailed *t*-test in (A, C). ns, not significant.


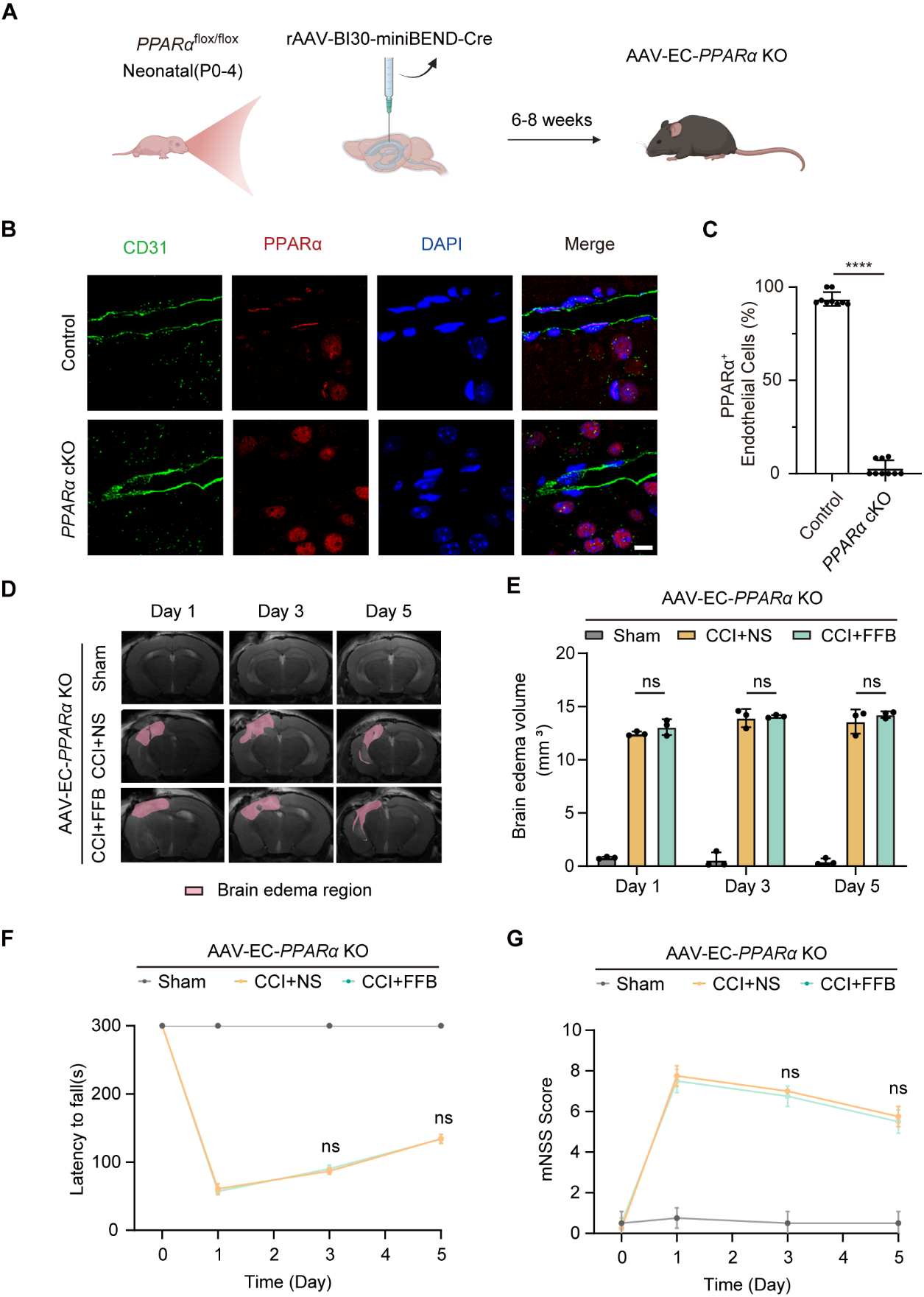


**Figure S5. FFB fails to attenuate brain edema in AAV-EC-*PPARα* KO mice.** (**A**) Schematic illustration of endothelial-specific *PPARα* KO generated by AAV-BI30-miniBEND-Cre injection in *PPARα*^flox/flox^ neonatal mice. (**B** and **C**) Representative immunofluorescence staining of CD31 (green), PPARα (red), and DAPI (blue) (B) and quantification of PPARα-positive endothelial cells (C) in control and AAV-EC-*PPARα* KO mice, scale bar = 10 μm. (**D** and **E**) Representative brain edema regions (pink) on MRI (D) and corresponding brain edema volumes (E) in sham, CCI with normal saline (NS), and CCI with FFB groups at days 1, 3, and 5 after injury in AAV-EC-*PPARα* KO mice. (**F** and **G**) Rotarod (F) and mNSS (G) tests in sham, CCI with NS, and CCI with FFB groups at days 1, 3, and 5 after injury in AAV-EC-*PPARα* KO mice. Error bars show mean ± SD. Statistical analysis was performed using unpaired two-tailed Student’s t-test in (C), one-way ANOVA with Tukey’s multiple comparison test in (E) and two-way ANOVA with Tukey’s multiple comparison test in (F, G). ns, not significant; ****, *p* < 0.0001.


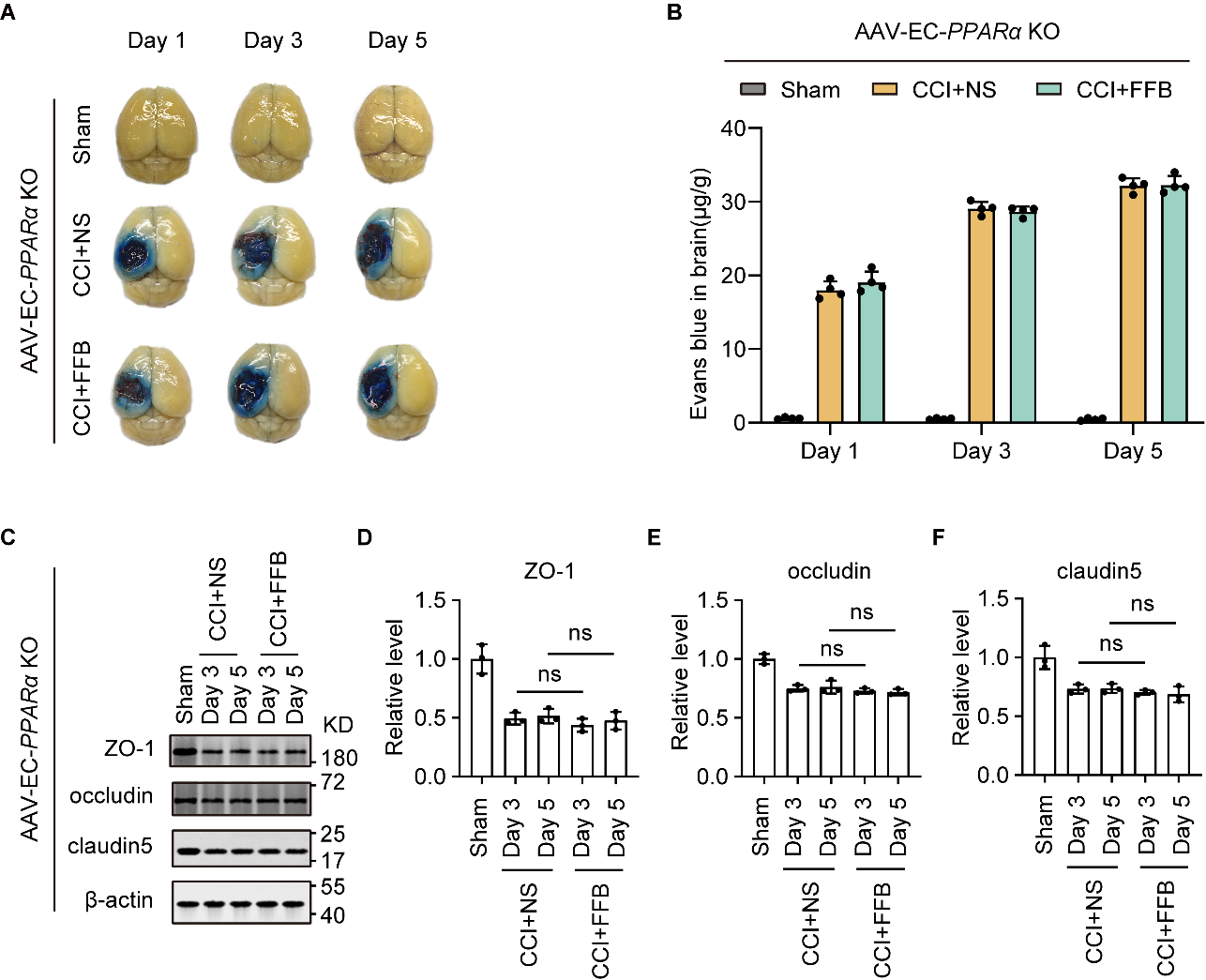


**Figure S6. FFB fails to preserve BBB integrity in AAV-EC-PPARα KO mice.** (**A**) Representative images of Evans blue extravasation in brains from sham, CCI with normal saline (NS), and CCI with FFB groups at days 1, 3, and 5 after injury in AAV-EC-*PPARα* KO mice. (**B**) Quantification of Evans blue content in brain tissue under the indicated conditions. (**C**-**F**) Representative western blotting showing ZO-1 (C), occludin (D), and claudin-5 (E) expression and corresponding quantification (F) in AAV-EC-*PPARα* KO mice under the indicated conditions. Error bars show mean ± SD. Statistical analysis was performed using one-way ANOVA with Tukey’s multiple comparison test in (B, D-F). ns, not significant.


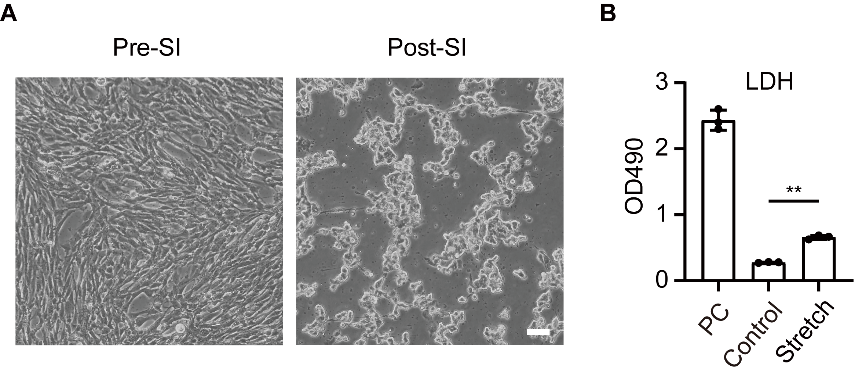


**Figure S7. Establishment of a severe SI model in bEnd.3 cells.** (**A**) The morphology of bEnd.3 cells before and after severely stretch-induced injury. Scale bar = 50 μm. (**B**) Lactate dehydrogenase (LDH) level in injured cells and their control. PC, positive control. Error bars show mean ± SD. Statistical analysis was performed using one-way ANOVA with Tukey’s multiple comparison test in (B). **, *p* < 0.01.


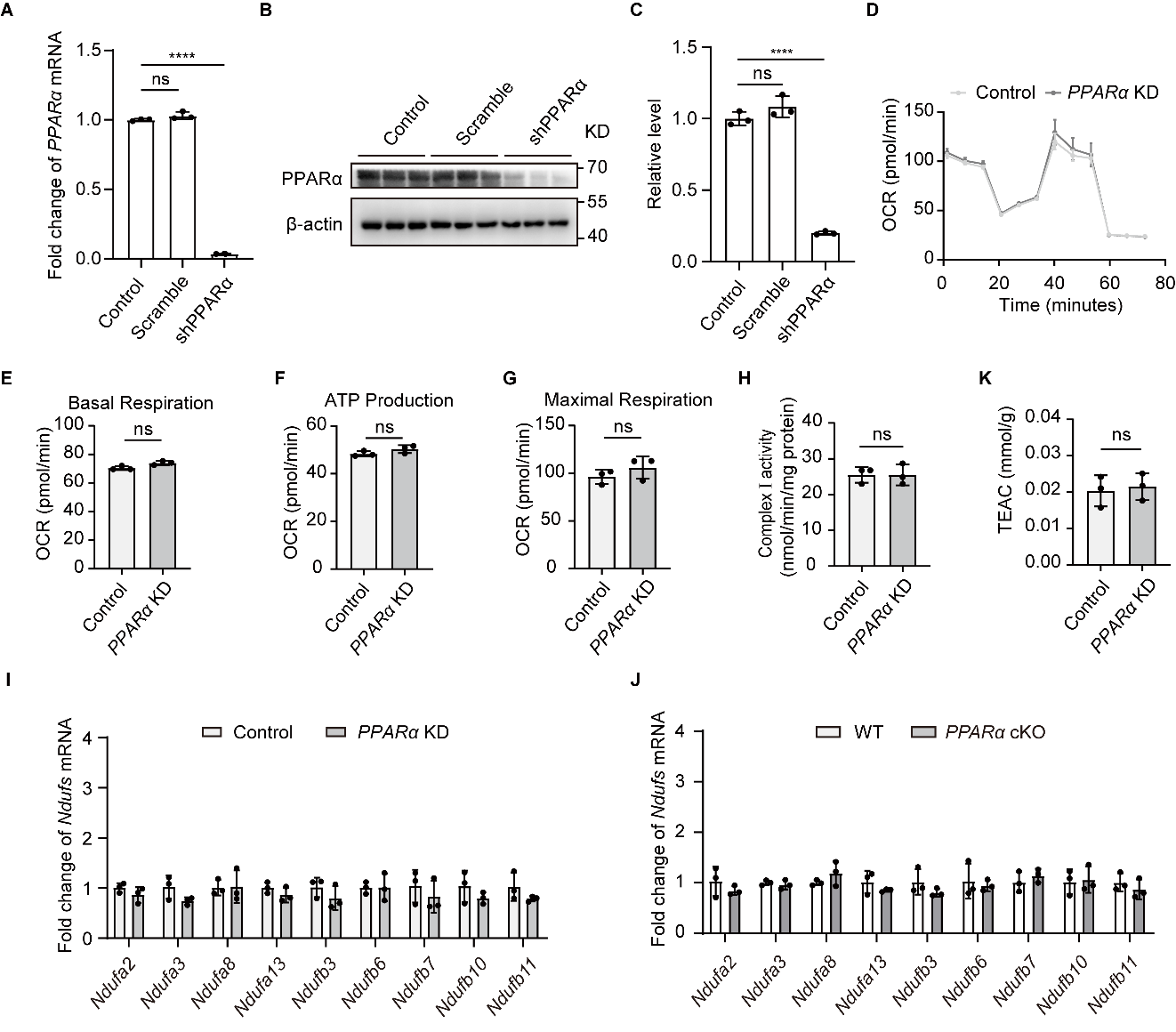


**Figure S8. PPARα knockdown does not affect OXPHOS without injury.** (**A**) Gene expression of *PPARα* in bEnd.3 cells transfected with scramble-shRNA and PPARα-shRNA (shPPARα). (**B** and **C**) Representative western blotting showing PPARα expression in bEnd.3 cells transfected with scramble-shRNA and PPARα-shRNA (shPPARα) (B). Quantification of PPARα in western blotting (C). (**D-G**) OCR analysis in *PPARα* KD cells and their control. Representative OCR trace (D); Quantification of basal respiration (E), ATP production (F), and maximal respiratory capacity (G). (**H**) Mitochondrail complex I activity in *PPARα* KD cells and their control. (**I**) Gene expression of *Ndufa* and *Ndufb* in *PPARα* KD cells and their control. (**H**) Gene expression of *Ndufa* and *Ndufb* in endothelial cells isolated from the brain of WT and *PPARα* cKO mice without CCI. (**K**) TEAC of *PPARα* KD cells and their control. Error bars show mean ± SD. Statistical analysis was performed using one-way ANOVA with Tukey’s multiple comparison test in (A, C) and unpaired two-tailed *t*-test in (E-K). ns, not significant; ****, *p* < 0.0001.


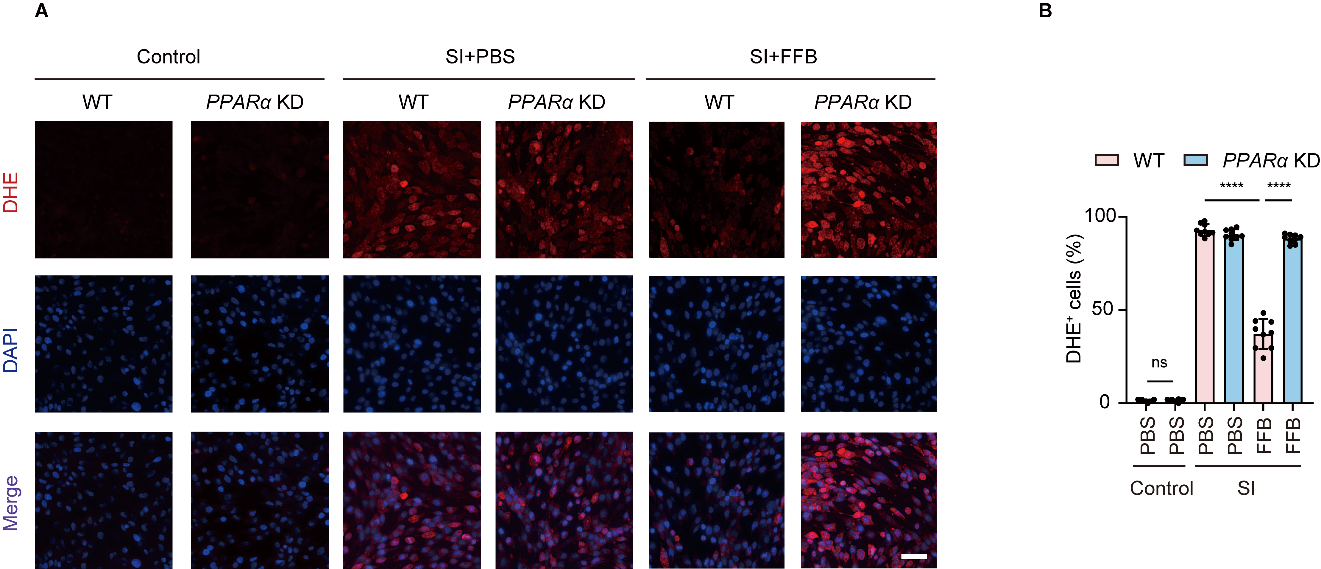


**Figure S9. FFB reduces ROS production in endothelial cells.** (**A**) Representative DHE fluorescence (red) in WT and *PPARα* KD bEnd.3 cells, without SI or following SI, treated with or without FFB as indicated. Scale bar = 50 μm. (**B**) Quantification of DHE positive cells in (A). Error bars show mean ± SD. Statistical analysis was performed using one-way ANOVA with Tukey’s multiple comparison test. ****, *p* < 0.0001.
